# Theta activity as a marker of cognitive development in infancy: A longitudinal study across the first two years of life

**DOI:** 10.1016/j.dcn.2025.101642

**Published:** 2025-11-04

**Authors:** Alicja Brzozowska, Johanna Ruess, Regina Ori Stoeckl, Martina Arioli, Stefanie Hoehl

**Affiliations:** a Department of Developmental and Educational Psychology, University of Vienna, Vienna, Austria; b Psychology, School of Social Sciences, Nanyang Technological University, Singapore; c Department of Psychology, University of Milano-Bicocca, Milan, Italy

**Keywords:** Infant, EEG, Theta, Cognitive development, Learning

## Abstract

Research shows that the theta rhythm in infant electroencephalogram indexes learning processes and is a promising candidate for a marker of early cognitive development. However, a scarcity of studies investigating the stability of individual differences in theta activity in infancy, and a large variability in analytical approaches in existing research constrain the interpretations of research findings. In our large longitudinal study, we related three different indices of frontocentral theta activity (absolute and relative power, and an index of theta modulation by novel content) at 6 and 12 months to cognitive development level, language skills, and visual attention at 24 months. We found an increase in theta power over the course of novel information encoding at 6 and 12 months, replicating prior studies. Both absolute and relative theta power, but not theta modulation index, showed a large degree of stability in individual differences from 6 to 12 months. Finally, absolute theta power at 6 and 12 months was a positive predictor of the general cognitive level, but not of specific skills (selective attention, language) at 24 months. Of note, we observed similar effects for absolute power in the alpha frequency band, suggesting that the effects are not specific to the theta frequency band. Our results support the involvement of the theta rhythm in cognitive development in infancy and point to absolute power as the potentially most sensitive index of individual differences in theta activity.

## Highlights

• Theta power increases over the course of a novel video viewing at 6 and 12 months.
• Individual differences in absolute and relative theta power are stable in infancy.
• Absolute theta power at 6 and 12 months positively predicts cognition at 24 months.

## 1 Introduction

The first two years of life are a time of rapid brain maturation, accompanied by remarkable changes in cognitive functioning (Alcauter et al., 2014, Bethlehem et al., 2022). Decades of research into infant electrical brain activity as measured with electroencephalography (EEG) have brought valuable insights into functional brain development during this transformational life period (Buzzell et al., 2023, Köster et al., 2023, Saby and Marshall, 2012). Recently, one particular EEG-derived neural signature has attracted special attention as a potentially powerful marker of early cognition – the theta oscillations (Begus and Bonawitz, 2020, Morales and Buzzell, 2025, Tan et al., 2024, Ünsal et al., 2024).

Theta oscillations span roughly between 4 and 8 Hz in the adult and between 3 and 6 Hz in the infant EEG (Orekhova et al., 1999). Although the generators of neural oscillations recorded from the scalp are not straightforward to identify, the EEG theta rhythm is generally attributed to result from activity in the cortical-hippocampal feedback loops (Lega et al., 2012). Accordingly, it is well established that the theta oscillations are involved in working memory processes in the adult brain (Sauseng et al., 2010). For instance, studies have shown that the power of the theta oscillations increases during encoding of novel information (Klimesch et al., 1994, Summerfield and Mangels, 2005), and that this increase is positively correlated with the amount of encoded information (Castro-Meneses et al., 2020, Jensen and Tesche, 2002).

Similar observations have been made in developmental populations. Meyer and colleagues (2019) found that in 4-year-olds, frontocentral theta power increased with prolonged engagement in a cognitively demanding task. These effects seem to be present even earlier in development, as for instance, 10-month-old infants were shown to exhibit an initial increase followed by a decrease in frontal theta power while viewing an attractive animated cartoon repeated several times (Piccardi et al., 2020). The power of the theta oscillations in the first years of life has also been shown to increase in contexts known to promote learning, such as violation of expectation paradigms (Berger et al., 2006, Köster et al., 2021), during object exploration (Begus et al., 2015, Michel et al., 2023), in response to gaze cueing (Angelini et al., 2023, Michel et al., 2015) and infant-directed actions (Meyer et al., 2023), and when expecting to receive information (Begus and Bonawitz, 2024). These findings suggest developmental continuity in the involvement of the theta rhythm in learning processes.

Remarkably, theta activity reflects not only within-individual engagement in novel information encoding, but it seems to also hold potential as an early marker of inter-individual differences in cognitive ability (Tan et al., 2024). Several recent studies used indices of theta activity recorded in infancy to successfully predict later performance in tasks measuring different aspects of cognition (Braithwaite et al., 2020, Brandes-Aitken et al., 2023, Jones et al., 2020, Rico-Picó et al., 2025, Sullivan et al., 2025, Tan et al., 2023). In 3-month-old infants, higher theta power during periods of sustained attention (as defined by a heart-rate measure) while watching dynamic social videos (‘Sesame Street’ video clips) was found to predict better recognition memory at 9 months (Brandes-Aitken, Metser, et al., 2023). Moreover, the degree to which theta power was modulated by novel content in a dynamic non-social video (scenes such as falling leaves, a plane flying across the sky, and similar) at 6 months was shown to positively relate to non-verbal cognitive level at 9 months (Braithwaite et al., 2020), and recorded at 12 months, to verbal and nonverbal cognition at 2, 3, and 7 years of age (Jones et al., 2020). Higher theta power during non-social video viewing (geometrical shapes in motion) and watching an experimenter blow bubbles at 9 months was also found to be positively related to cognitive flexibility at 16 months (Rico-Picó et al., 2025). On the other hand, one study found a negative relation between theta power during non-social video viewing (colorful objects in motion) and concurrent parent-reported attention in infants between the ages of 6 and 12 months (Perone and Gartstein, 2019), while another demonstrated that higher theta power during passive viewing of a spinning bingo wheel recorded between 22 and 42 months predicted lower intelligence as late as at 18 years (Tan et al., 2023).

One explanation for the differing cognitive correlates of theta activity during development concerns the conditions under which EEG was recorded. A recent review suggested that resting-state and task-related theta activity in developmental populations have diverging correlates: while increased theta activity during resting-state negatively correlates with cognitive abilities, increased task-related theta activity is associated with higher cognitive performance (Tan et al., 2024). This divergence was attributed to increased resting-state theta power potentially indicating a developmental lag in brain maturation, a process reflected in the gradual shift of EEG activity toward higher frequencies, whereas higher task-related theta power is thought to signify the engagement of cognitive processes (see Tan et al. 2024 for a detailed discussion of this argument). While this reasoning appears to be well supported by studies with children aged 3 years and above (e.g. Liu et al., 2014; Lum et al., 2022; Maguire and Schneider, 2019; Morales et al., 2023), it has limitations in the case of infants, with whom “true” resting-state recordings (i.e., eyes-open/eyes-closed without stimulation) are not feasible. Indeed, passive viewing of dynamic non-social stimuli (e.g. colorful moving objects in a video) was labeled as a resting-state condition in some studies with infants (e.g. Perone and Gartstein, 2019; Tan et al., 2023), and in others as a task (e.g. Braithwaite et al., 2020; Jones et al., 2020). The degree to which the presented stimuli are cognitively engaging to the infant might therefore determine how comparable the passive viewing conditions are to the eyes-open/eyes-closed resting-state conditions used with older populations. This might explain why e.g. Tan et al. (2023) found cognition to be negatively related to theta activity during passive viewing of a spinning bingo wheel (presumably a relatively unengaging stimulus for 22–42 month olds – more comparable with resting-state), while Rico-Picó et al. (2025) found cognition to be positively related to theta activity during observation of a person blowing soap bubbles (highly engaging stimulus for 9-month-olds – more comparable with a task).

In infant studies, where even the ‘resting-state’ conditions involve presentation of novel stimuli and are therefore often associated with a degree of cognitive load, another potentially significant source of discrepancies in findings is worth consideration: the fact that different studies used different indices of theta activity. The most common indices include absolute theta power (e.g. Brandes-Aitken, Metser, et al., 2023), relative theta power, calculated as a ratio of average theta power with respect to the average power across all frequencies (e.g. Perone and Gartstein, 2019), and theta modulation indices, calculated as a correlation between a time series of absolute theta power values during task engagement and a corresponding time index vector (e.g. Braithwaite et al., 2020). Arguments have been made in favor of each of these indices in research on individual differences.

Absolute power is a direct measure of electrical activity, used in many seminal studies (e.g. Bell and Fox, 1992; Orekhova, 1999). It is also best described in terms of its developmental trajectories, with studies consistently reporting an increase in absolute theta power (measured during passive viewing of non-social stimuli) in the first year of life (Gabard-Durnam et al., 2019, Jing et al., 2010, Wilkinson et al., 2023). Importantly, absolute power quantifies power within a predefined frequency band independent of activity in other frequency bands. In case of low frequency rhythms such as the theta rhythm, this measure may thus be relatively unaffected by artifacts manifesting in high frequencies (> 20 Hz), such as muscle artifacts (Muthukumaraswamy, 2013), commonly present in infant EEG. Studies with infants using absolute theta power (measured during exposure to novel stimuli) predominantly find positive associations with cognition (e.g. Brandes-Aitken, Metser, et al., 2023; Orekhova et al., 1999; Wass et al., 2018; Xie et al., 2018).

On the other hand, relative power conveys information about the contribution of a given frequency band to the overall EEG power spectrum. As brain maturation is reflected by a relative shift from lower to higher frequency bands, in case of the theta frequency band, one would expect relative power (especially in contexts with low cognitive load) to negatively correlate with cognition (Tan et al., 2024). Indeed, a number of infant studies reported negative associations between relative theta power and cognition (Perone and Gartstein, 2019; Tan et al., 2023). Moreover, some have argued that relative power is, in comparison with absolute power, less affected by factors such as bone thickness or skull impedance, which change over development (Benninger et al., 1984).

Finally, the theta modulation index quantifies theta power change during task engagement. As an increase in theta power has been linked to information encoding (e.g. Begus et al., 2015; Jones et al., 2020), the theta modulation index might capture working memory-related processes best. Higher, positive values of the theta modulation index would therefore indicate stronger modulation of the theta activity by novel content, reflecting stronger cognitive engagement. It has also been argued that changes in theta activity during task engagement may be particularly informative about oscillatory (vs. aperiodic) activity (Tan et al., 2024). Theta modulation during exposure to novel non-social stimuli in early infancy was shown to positively predict later cognition (Braithwaite et al., 2020, Jones et al., 2020).

Of note, studies linking different indices of theta activity to cognitive performance also differed in cognitive load during theta activity measurement. For instance, many studies showing positive correlations between absolute theta power and cognition included clear definitions of engagement events (e.g., theta was measured prior to visual fixations, Wass et al., 2018) or incorporated additional measures of cognitive engagement (e.g. heart-rate defined sustained attention, Brandes-Aitken, Metser et al.-, 2023; Xie et al., 2018). In contrast, research demonstrating negative associations of relative theta power with cognition did not incorporate such events or additional measures (Perone and Gartstein, 2019, Tan et al., 2023). This lack of consistency in task features makes it difficult to disentangle whether the direction of associations between theta activity and cognitive performance was driven by the experimental procedure (‘resting-state’ vs. task engagement) or analytical choices (e.g., absolute vs. relative power).

In addition to a lack of clarity as to which index of theta activity might be best suited as a marker of early cognition, there are some open questions regarding the stability of individual differences (degree of consistency over time) in theta activity indices in the first years of life (Begus and Bonawitz, 2020). On a group level, the developmental trajectories of absolute and relative theta power (measured during passive viewing of nonsocial stimuli) in the first year and a half seem to diverge, with the former increasing in the first year (Gabard-Durnam et al., 2019, Jing et al., 2010, Rico-Picó et al., 2023, Wilkinson et al., 2023, Wilkinson et al., 2024) and the latter decreasing (Rico-Picó et al., 2023). However, less is known about the within-individual stability of theta activity in early infancy, with some reports of relative theta power being more stable than absolute theta power (Rico-Picó et al., 2023), but no studies to date examining the stability of the theta modulation index. As a robust index of cognitive ability is likely to exhibit a degree of within-individual stability in individual differences across development, characterizing this property of infant theta activity appears crucial.

Plenty of research so far implicates the involvement of theta activity in cognitive development across infancy, but open questions remain. The present study examined the association between frontocentral theta activity at 6 and 12 months, and cognitive development level at 24 months. Firstly, we aimed to replicate previously reported findings indicating that frontocentral theta power in infants increases over the course of viewing novel stimuli, suggesting theta’s involvement in working memory processes. Secondly, we set out to take a closer look at the index of theta modulation by novel content, its reliability, and longitudinal stability in comparison with more established indices of theta activity such as absolute and relative power. Finally, we explored the predictive relations between indices of frontocentral theta activity at 6 and 12 months and cognitive development level, visual attention, and language at 24 months. To verify whether the examined effects are specific to the theta frequency band, we also performed the same analyses with indices of activity in the alpha frequency band (6 – 9 Hz).

## 2 Materials and methods

The study is a part of a large longitudinal project investigating neural correlates of early cognitive development. Of note, some results from the project (pertaining to alpha activity during video viewing and performance in a predictive cueing task at 6 months – no overlap with the current investigation) have already been published (Arioli et al., 2024). The hypotheses regarding the associations between the theta modulation indices at 6 months and 12 months and the cognitive outcomes at 24 months were included in the preregistration document on the platform AsPredicted: https://aspredicted.org/wvgh-n7xz.pdf (point 8). The remaining analyses presented here were not preregistered. The analysis scripts and data supporting the results of the study are openly available at https://github.com/alicja444/ThetaActivity and https://osf.io/63ena/, respectively.

### 2.1 Participants

**Fig. 1:**
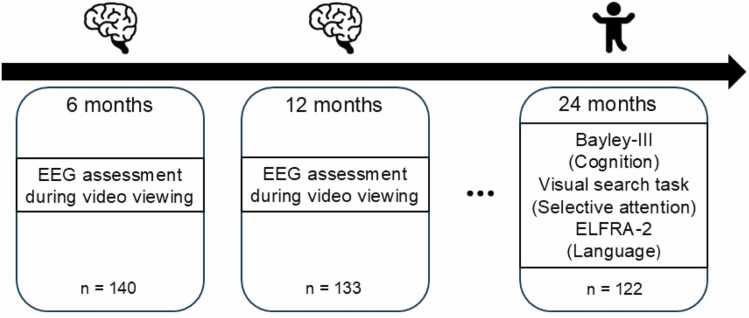
Study design.

The infants enrolled in the study were full-term, typically developing 6-month-olds (mean age = 199 days, standard deviation = 9 days). At the first time point (6 months) one hundred forty (n = 140, 76 female) infants participated in the study. One hundred thirty three (n = 133, 72 female) of them returned at the second time point, 12 months (mean age = 378 days, standard deviation = 13 days), and one hundred twenty two (n = 122, 65 female) at the third time point, 24 months (mean age = 740 days, standard deviation = 36 days). The longitudinal design of the study is depicted in Fig. 1.

Demographic information and descriptive statistics of the sample are presented in Table 1. Cultural affiliation and birth country were assessed using an open-ended question in a background and demographics questionnaire. The grouping of these variables was adapted from Vietze et al. (2022). Given the presence of self-reported European cultural affiliation among Austrian natives, bicultural Austrians and other Europeans, we introduced a new category, “European Identification”.

**Table 1:** Demographic information and descriptive statistics of the sample.

Parameters		Children (N = 140)		
		N(%)		M(SD)
Gestational age [weeks]		138 (99 %)		39.7 (1.3)
Missing		2 (1 %)		
Language household				
Monolingual		59 (42 %)		
Multilingual		43 (31 %)		
Missing		38 (27 %)		
Annual household income				
< 26.000 EUR		5 (4 %)		
26.000 – 45.000 EUR		17 (12 %)		
> 45.000 EUR		88 (63 %)		
Missing		30 (21 %)		
Age at birth: primary caregiver [years]		118 (84 %)		34 (4)
Missing		22 (16 %)		
Education: primary caregiver				
Upper Secondary Education		19 (13 %)		
Tertiary Education		99 (71 %)		
Missing		22 (16 %)		
Cultural affiliation: primary caregiver				
Only Austrian Identification		70 (50 %)		
Austrian Bicultural Identification		17 (12 %)		
European Identification		8 (6 %)		
Non-Austrian Identification		19 (14 %)		
Missing		26 (18 %)		
Birth region: primary caregiver				
Austria		79 (56 %)		
West Europe		20 (14 %)		
East Europe		14 (10 %)		
Other		2 (2 %)		
Missing		25 (18 %)		
Age at birth: secondary caregiver [years]		116 (83 %)		37 (5)
Missing		24 (17 %)		
Education: secondary caregiver				
Lower Secondary Education		2 (1 %)		
Upper Secondary Education		33 (24 %)		
Tertiary Education		81 (58 %)		
Missing		24 (17 %)		
Cultural affiliation: secondary caregiver				
Only Austrian Identification		63 (45 %)		
Austrian Bicultural Identification		18 (13 %)		
European Identification		4 (3 %)		
Non-Austrian Identification		26 (18 %)		
Missing		29 (21 %)		
Birth region: secondary caregiver				
Austria		76 (55 %)		
West Europe		25 (18 %)		
East Europe		6 (4 %)		
Other		6 (4 %)		
Missing		27 (19 %)		
		

The sample size was calculated based on estimated effect sizes for other effects investigated in this project (the discussion of which goes beyond the scope of the present manuscript). The study received approval of the Ethics Committee of the University of Vienna (ref. 00737). Before participation, informed written consent was obtained from the primary caregiver at each timepoint. The study was conducted in line with the principles of the Declaration of Helsinki.

### 2.2 Stimuli and procedure: 6 months & 12 months

#### 2.2.1 Non-social video

The participants watched a non-social video (taken from Braithwaite et al., 2020) for up to 2 min and 14 s. The video consisted of several short clips depicting scenes such as falling leaves, a plane flying across the sky, basketball balls bouncing on the floor, and similar. The non-social video was accompanied by auditory white noise.

#### 2.2.2 Procedure

The procedure was identical at 6 and 12 months. The testing session took place in a room adapted for EEG data collection, at the Vienna Children’s Studies lab (Faculty of Psychology, University of Vienna). After the study procedure was explained to the caregiver and written informed consent was collected, the infant’s head circumference was measured and an appropriately sized EEG cap with electrodes was placed on the infant’s head. During the viewing of the non-social video, the infant was seated in the caregiver’s lap, approximately 60 cm from the screen. The infant was presented with three tasks, in a fixed order: the non-social video, a Gaze Cue task, and a Predictive Cueing task (the latter two not relevant to the present investigation). The tasks were displayed on an Iiyama G-Master GB2560HSU screen with a 144 Hz refresh rate, and the accompanying audio was presented using Logitech Multimedia Z200 speakers. Two Axis P1365 Mk II cameras recorded the screen and infant looking behavior towards the screen at 25 frames per second. The family’s visit in the lab lasted about 1 h, about 10 min of which were the testing session in front of the screen. The family’s travel costs to the lab were reimbursed, and the baby received an age-appropriate toy and a certificate of participation.

#### 2.2.3 Looking behavior data

Infant looking behavior towards the screen (on/off) was coded offline with the use of the Interact software (Mangold, 2018). Out of the total of 140 videos, 25 videos were coded by two independent raters, frame by frame, yielding excellent inter-rater reliability (κ = 0.92).

#### 2.2.4 EEG data collection

ActiCap EEG caps (EasyCap GmbH) of appropriate sizes (42–48 cm circumference) were used in data collection, in combination with an active 32 Ag/AgCl electrode set (Brain Products GmbH) and conductive gel. We aimed to keep the impedances below 10 kΩ whenever possible. The placement of the electrodes was an adapted 10–20 layout system, with the online reference electrode positioned behind the left ear (TP9), and the ground electrode in the front (Fp1). One electrode was placed underneath the infant’s right eye with the purpose of capturing electrooculography (EOG) signal for tracking eye movements. However, many infants did not tolerate this well, which led to either missing or noisy EOG data, forcing us to eventually discard this channel in our analyses. BrainAmp DC amplifier (Brain Products GmbH) was used for EEG signal recording, with a sampling frequency of 500 Hz. The amplifier was connected to a BrainProducts TriggerBox device, which synchronized triggers sent from the presentation computer and the EEG/video recording computer. BrainVision Recorder (BrainVision Recorder, Vers. 1.24.001, Brain Products GmbH, Gilching, Germany) software was used for EEG signal recording.

#### 2.2.5 EEG data preprocessing

The EEG data was preprocessed with the use of the standardized HAPPILEE pipeline for lower density recordings (Lopez et al., 2022) in combination with custom MATLAB (The MathWorks Inc, 2022) scripts, and functions from the EEGLAB toolbox (Delorme and Makeig, 2004). Firstly, line noise processing was conducted with a multi-taper regression approach to eliminate the 50 Hz electrical noise artifact. Next, a bandpass zero-phase Hamming-windowed sinc FIR filter with cut-off frequencies 1 and 100 Hz was applied to the continuous data. Next, artifact correction was performed with the use of wavelet thresholding. The data was then segmented into 1-second epochs. Channels deemed artifact-contaminated according to FASTER criteria (variance, median gradient, amplitude range, and deviation from mean amplitude; (Nolan et al., 2010) were interpolated with spherical splines within epochs (up to 3 channels, otherwise the epoch was rejected). Next, individual epochs were rejected based on a combination of amplitude-based (signal below/above +/- 150 μV) and joint-probability (considering how likely an epoch’s activity was given the activity of other epochs for that same channel, as well as other channels’ activity for the same segment) criteria. Indices of quality assessment of the pipeline performance were inspected, and participants for whom the cross-correlation values across all frequencies before and after wavelet thresholding were below 0.1 (indicating dramatic signal change during waveleting) were excluded from further analyses (Monachino et al., 2022; see Table S1 in Supplementary Materials for the number of excluded participants at each timepoint). The data was then re-referenced to average reference. Finally, segments where infants’ gaze was coded as directed away from the screen were rejected.

#### 2.2.6 EEG data analysis: theta activity indices

In line with previous literature (e.g. Braithwaite et al., 2020; Brandes-Aitken, Metser, et al., 2023), analyses of theta activity focused on frontocentral channels (F3, Fz, F4, FC3, FCz, and FC4) and the 3 – 6 Hz frequency range. At both 6 and 12 months, a minimum of 10 clean segments was required for a participant to be included in the analysis (see Table 2 for mean numbers of segments included in each age group).

**Table 2:** Descriptive statistics for the EEG measures at 6 and 12 months, and the cognitive outcomes at 24 months.

	n	Mean	SD	Minimum	Maximum
Number of clean segments at 6 m	98	64	16	15	91
Number of clean segments at 12 m	87	53	20	10	102
Absolute theta power [μV^2^] at 6 m	98	9.01	0.95	6.97	11.27
Absolute theta power [μV^2^] at 12 m	87	9.67	1.02	6.69	12.60
Relative theta power at 6 m	98	0.50	0.06	0.35	0.61
Relative theta power at 12 m	87	0.46	0.05	0.31	0.55
Theta modulation score at 6 m	98	0.20	0.19	−0.23	0.64
Theta modulation score at 12 m	87	0.13	0.21	−0.39	0.61
Bayley-III Cognitive Index at 24 m	117	67	5	55	76
Selective Attention score at 24 m	111	3.38	1.33	0.33	6.67
ELFRA−2: expressive language score at 24 m	115	129	59	4	232
ELFRA−2: syntax score at 24 m	115	25	13	0	53
ELFRA−2: morphology score at 24 m	115	6	4	0	16

Absolute theta power was estimated on data segmented into 1-second-long epochs with 50 % overlap, applying a Fast Fourier Transform with a 1-second Hamming window. The power spectral density was calculated for each channel, then log-transformed and averaged across channels. Absolute theta power was then obtained by integrating the power spectral density over the theta frequency range.

Relative theta power was calculated as absolute power in the theta frequency band averaged across the electrodes of interest divided by total absolute power in the 1 – 45 Hz frequency range (similar to e.g. Perone and Gartstein, 2019; Tan et al., 2023) averaged across the electrodes of interest (not applying log-transformation).

Theta modulation index was calculated closely following the approach described by Braithwaite et al. (2020). Theta power for each clean (non-overlapping) segment (1 s) was calculated using Fast Fourier Transform and averaging across the electrodes of interest. Next, Pearson correlation was calculated between the theta power and the segment number, with the resulting coefficient indexing the theta power change over the course of the video for each participant.

In order to check for the specificity of the investigated effects to the theta frequency band, analogous indices were computed for the alpha frequency band (6 – 9 Hz).

### 2.3 Stimuli and procedure: 24 months

#### 2.3.1 Visual search task: selective attention

The visual search task (Mulder et al., 2014) was administered to assess the child’s visual selective attention skills at 24 months. The task was presented on a Lenovo ThinkPad T460 14” laptop screen using the ePrime 3.0. software (Psychology Software Tools, 2016). The task consisted of three trials in which children were presented with displays of 48 cartoon drawings of bears, donkeys, and elephants on a 6 × 8 grid, and were asked to locate and point to as many targets (elephants) among distractors (bears and donkeys) as possible, within 40 s (see Mulder et al., 2014 for a detailed description of the task). The Selective Attention score was the average number of targets pointed to across trials (range: 0 – 8). Following Mulder et al. (2014), an average of 0 across all trials was treated as a missing value, as it indicated a possibility that the child did not understand the task instructions. The task was administered by trained experimenters and filmed with a GoPro HERO8 camera. The video recordings of the task were reviewed in rare cases when the experimenters were not certain whether participant responses were correctly recorded (e.g. the child pointed to a target shortly before trial end). Previous research using the Visual search task (Veer et al., 2017) has demonstrated its very good internal consistency at 2.5 years (Cronbach’s α =.88) as well as significant stability across a 6-month period (r = .59, p < .01, from 2.5 to 3 years).

#### 2.3.2 The Bayley scales of infant and toddler development – third edition: cognitive index

The Bayley Scales of Infant and Toddler Development – Third Edition (Bayley-III), the German standardized version (Bayley et al., 2014) was used to assess children’s developmental outcomes at 24 months. The Bayley-III scales include subscales of cognitive, receptive and expressive language, as well as fine and gross motor development. Prior to the beginning of data collection, the experimenters involved in the Bayley-III administration participated in in-depth training on the use of the tool led by two highly experienced clinical psychologists working at the Vienna General Hospital, which was followed by an extended period of practical exercises. In our study, we only administered the cognitive and gross motor subscales. In the present investigation, we use the Cognitive Index (composite score) as a measure of the child’s cognitive development, capturing cognitive dimensions including information processing, processing speed, concept formation, and categorization. The internal consistency of the German Bayley-III Cognitive Index assessed with split-half reliability method was 0.72, indicating high reliability (Bayley et al., 2014).

#### 2.3.3 ELFRA-2: language development

Caregivers were asked to fill out the ELFRA-2 questionnaire (Grimm and Doil, 2006), which assesses infant expressive language skills (a list of 260 words), syntax (25 questions) and morphology (11 questions) usage, in German. The internal consistency of the subscales ranges from 0.84 to 0.98, indicating high to very high reliability (Grimm and Doil, 2006).

#### 2.3.4 Procedure

At 24 months, the infants and their caregivers returned to the Vienna Children’s Studies lab to complete a battery of tasks assessing various aspects of their development. Additionally, caregivers were asked to fill out a short demographic questionnaire, and the ELFRA-2 questionnaire about the child’s language skills. After informing the caregivers about the study procedure, signed consent forms were collected. Next, a brief warm-up phase occurred, during which the child engaged in playing with the experimenter. This phase was designed to help the participants acclimate to the study and enhance their compliance. After the warm-up phase, the participants were presented with the following tasks, in a fixed order: Drumming task, assessing sensorimotor synchronization (not analyzed in the present study), Visual search task, assessing visual selective attention, and the cognitive and gross motor subscales of the Bayley-III scales. Finally, at the end of the visit, we collected the questionnaires from the caregiver. The entire visit to the lab lasted around 2.5 h. At the end of the visit, the caregiver received a travel cost reimbursement, a canvas bag, and a choice between a popular science book about child development and a €15 voucher to a department store. The child received an age-appropriate toy, and a certificate of participation.

#### 2.3.5 Analytic strategy

The analytic plan was divided into four parts. First, we examined the properties of the relatively less established index of theta activity, the theta modulation index. We tested whether we could replicate prior findings showing that theta power increases over the course of video viewing at 6 and 12 months by conducting one-sample *t*-tests on theta modulation indices against the value of zero (following Braithwaite et al., 2020) at both age points. Additionally, we examined the reliability of the theta modulation index through an adapted split-half reliability calculation approach (Xu et al., 2024), whereby we calculated intra-class correlation coefficients between theta modulation indices calculated on even- and odd-numbered segments separately (Meyer et al., 2014). Next, we investigated the development and stability of individual differences in theta activity in the first year of life by calculating paired-samples *t*-tests, Pearson correlations and intra-class correlations within mixed-effects models between the values of absolute power, relative power and modulation indices of theta activity at 6 and 12 months. We then investigated whether indices of theta activity could predict subsequent cognitive development using a stepwise (both forward and backward) algorithm implemented as a stepAIC() function in the R package MASS (Venables and Ripley, 2002) on linear regression models including relevant predictors. The algorithm was used to automate the process of stepwise model selection by iteratively adding and removing predictors to find the model with the best fit, as assessed with the Akaike Information Criterion (AIC). Finally, to determine whether the observed effects were specific to the theta frequency band, we repeated the above-described steps for indices of alpha (6–9 Hz) activity.

## 3 Results

Seventy percent (70 %) of participants at 6 months and sixty five percent (65 %) of participants at 12 months contributed valid EEG data. Descriptive statistics of the variables of interest can be found in Table 2. Detailed information regarding the reasons for missingness of data for each variable at each timepoint is available in Supplementary Material (Table S1).

Exploring the theta modulation index

To verify whether we can replicate the prior findings with 6-month-olds (Braithwaite et al., 2020), 10-month-olds (Piccardi et al., 2020) and 12-month-olds (Jones et al., 2020) showing overall increase in theta power over the course of video viewing, we conducted one-sample *t*-tests on theta modulation indices against the value of zero. Both at 6 months (t(97) = 10.66, p < 0.001, d = 1.08), as well as at 12 months (t(86) = 5.60, p < 0.001, d = 0.60) the theta modulation indices were significantly larger than zero. These results indicate that on average, theta power increased over the course of video viewing at both timepoints, in agreement with prior research. Additional analyses demonstrating the good fit of the linear model to grand average theta power data at both timepoints are presented in Supplementary Materials (S10).

One of the objectives of the present investigation was to examine the reliability of the theta modulation index at 6 and 12 months. To do so, we adapted a split-half reliability calculation approach (Xu et al., 2024) by calculating two separate theta modulation indices for each infant: one for the even-numbered segments, and one for the odd-numbered segments (similar to e.g. Meyer et al., 2014). Next, following prior investigations into the reliability of infant EEG measures (Noreika et al., 2020), we calculated intraclass correlation coefficients between the two indices. At 6 months, the theta modulation index had fair to substantial reliability (95 % confidence interval for ICC = [0.30 – 0.61], and at 12 months – poor to moderate reliability (95 % confidence interval for ICC = [0.07 – 0.45]).

Development and stability of theta activity in the first year of life

**Fig. 2:**
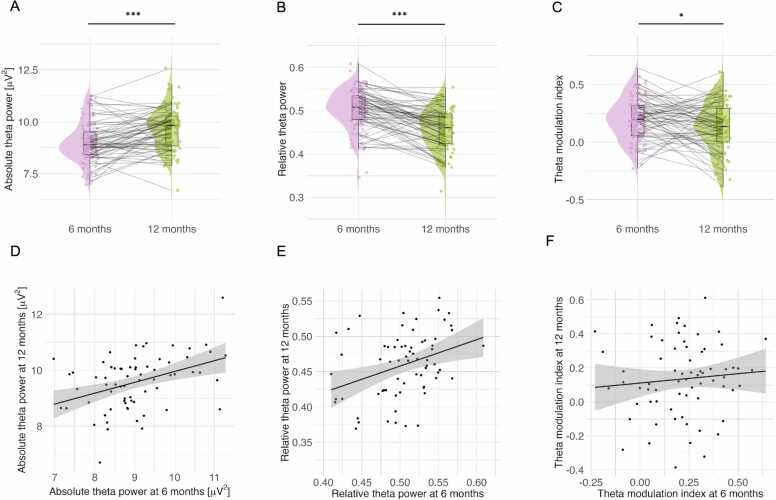
The development of theta activity indices from 6 to 12 months. (A) Absolute theta power at 6 and 12 months; (B) Relative theta power at 6 and 12 months; (C) Theta modulation index at 6 and 12 months (D) Relation between absolute theta power at 6 and 12 months; (E) Relation between relative theta power at 6 and 12 months (F) Relation between theta modulation index at 6 and 12 months.

The developmental trajectories of absolute theta power, relative theta power, and theta modulation from 6 to 12 months are shown in Fig. 2. Paired samples *t*-tests (on a subset of participants who had valid EEG data at both timepoints, n = 68) revealed that on a group level, absolute theta power increased from 6 to 12 months (t(67) = 4.19, p < 0.001), relative theta power decreased from 6 to 12 months (t(67) = -7.36, p < 0.001), and theta modulation index decreased from 6 to 12 months (t(67) = -2.58, p = 0.012). While group-level trends were found for all three indices of theta activity, inter-individual variability in how these measures changed from 6 to 12 months is evident in the plots, particularly for the theta modulation index (see Fig. 2, panels A, B, and C).

To examine the stability of theta activity indices, we conducted Pearson correlations relating the measures at 6 months to the measures at 12 months. We found that absolute theta power at 6 and 12 months was moderately correlated (r(66) = 0.402, p < 0.001; Fig. 2 panel D). Similarly, we found a significant moderate correlation between relative theta power values at 6 and 12 months (r(66) = 0.351, p = 0.003; Fig. 2 panel E). However, the theta modulation index was not correlated at 6 and 12 months (r(66) = 0.09, p = 0.445, Fig. 2 panel F).

As a secondary stability assessment, we calculated ICC values within mixed-effects models (including random effects of participants) for the three indices of theta activity between 6 and 12 months. The values were higher for absolute theta (ICC = 0.40) and relative theta (ICC = 0.39) than for theta modulation (ICC = 0.10).

Predicting cognitive outcomes with theta activity in the first year of life

The next step in our analysis was to evaluate whether we can predict cognitive development at 24 months using theta activity in the first year of life. As we did not have specific hypotheses regarding different aspects of language development, we calculated a composite Language score as an average of standardized values of ELFRA-2 subscales of expressive language, syntax, and morphology. Therefore, we ended up with three outcome variables: Bayley-III Cognitive Index, Selective attention score, and Language. Additionally, as not all participants were tested at exactly 24 months (Mean age = 744 days, SD = 18 days, Min. = 695, Max = 813) due to various difficulties related to scheduling (not atypical for longitudinal studies), we included a predictor variable *age at outcome* (in days) in all the analyses.

In order to examine which indices of theta activity in the first year of life are best predictors of later cognitive development, we fitted several linear regression models to the data, and selected the best model for each outcome variable using a stepwise (both backward and forward) algorithm based on Akaike Information Criterion (AIC), implemented as a *stepAIC()* function in the R package *MASS* (Venables and Ripley, 2002). Multicollinearity was assessed using the Variance Inflation Factors (VIF), which were all below 1.03, indicating no multicollinearity issues. Following Tan et al. (2023), and to include as much data as possible in this analysis, we calculated composite scores by averaging, for each participant, standardized scores of absolute theta power, relative theta power, and theta modulation across both timepoints (6 and 12 months). For participants who only had data at one of the two timepoints, the score comprised only of data from that timepoint. The full model for each outcome variable included *age at outcome*, *composite absolute theta power*, *composite relative theta power*, and *composite theta modulation index* as predictors. Details about the models considered (but rejected) by the stepwise algorithm are presented in Supplementary Materials (S9).

**Fig. 3:**
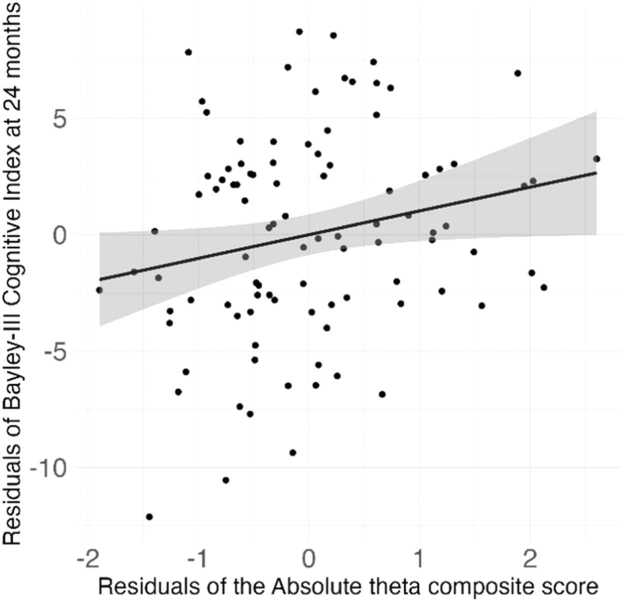
Partial regression plot of the adjusted relationship between Composite absolute theta power and Bayley-III Cognitive Index scores (standardized β = 0.21, 95 % CI [0.01, 0.40], t = 2.09, p = 0.039).

For the Bayley-III Cognitive Index, the best model included two predictors: *composite absolute theta power* and *age at outcome* (F(2, 93) = 4.395, p = 0.015), explaining 8.6 % of the variance in the outcome variable (R^2^ = 0.086, adjusted R^2^ = 0.067). *Composite absolute theta power* was significantly positively associated with the Bayley-III Cognitive Index scores (standardized β = 0.21, 95 % CI [0.01, 0.40], t = 2.09, p = 0.039), and so was *age at outcome* (standardized β = 0.20, 95 % CI [0.00, 0.40], t = 1.996, p = 0.049). The adjusted relation between the *composite absolute theta power* scores and the Bayley-III Cognitive scores at 24 months, controlling for *age at outcome*, is depicted in Fig. 3.

The best-fitting model predicting Selective attention scores included only the intercept, indicating that none of the included variables meaningfully predicted the dependent variable. The intercept of the model was 3.49 (SE = 0.136, t = 25.57, p < 0.001), with a residual standard error of 1.301 on 90 degrees of freedom.

For Language, the best-fitting model only included *age at outcome* as a predictor (F(1, 92) = 7.039, p = 0.009), explaining 7.1 % of the variance (R^2^ = 0.071). Age at outcome positively predicted Language scores (standardized β = 0.27, 95 % CI [0.07, 0.47], t = 2.653, p = 0.009). None of the indices of theta activity in the first year of life were predictive of language outcomes at 24 months.

In order to explore whether indices of theta activity at either timepoint (6 months or 12 months) were particularly predictive of later cognitive outcomes, we repeated the above-described procedure of model fitting without calculating composite scores but including the 6 month and 12 month variables as separate predictors, on a subset of participants with valid EEG data at both timepoints. Here, the full model for each outcome variable included the following predictors: *age at outcome*, *absolute theta power at 6 months*, *absolute theta power at 12 months*, *relative theta power at 6 months*, *relative theta power at 12 months*, *theta modulation index at 6 months*, and *theta modulation index at 12 months*.

The best-fitting model predicting the Bayley-III Cognitive Index included *theta modulation index at 6 months*, *absolute theta power at 6 months,* and *age at outcome* as predictors (F(3, 55) = 2.92), and was marginally significant (p = 0.042), explaining 13.7 % of the variance in the dependent variable (R^2^ = 0.137, adjusted R^2^ = 0.090). *Theta modulation index at 6 months*, was a marginally non-significant positive predictor of the Bayley-III Cognitive Index scores (standardized β = 0.24, 95 % CI [-0.01, 0.49], t = 1.929, p = 0.058). *Absolute theta power at 6 months* (standardized β = 0.20, 95 % CI [-0.06, 0.45], t = 1.562, p = 0.124) and *age at outcome* (standardized β = 0.18, 95 % CI [-0.07, 0.43], t = 1.439, p = 0.156) were not significant predictors. In the initial model, we included both 6- and 12-month predictors, which restricted the sample to participants with Bayley-III scores at 24 months and usable EEG data at both timepoints (n = 59). However, because the best-fitting model included only 6-month predictors, we were able to rerun it in a larger sample that included all participants with Bayley-III scores and usable 6-month EEG data (n = 81), regardless of whether they also had 12-month data. This allowed us to verify whether the result would still hold with increased sample size.

In this larger sample, the model was marginally significant (F(3, 77) = 4.303, p = 0.047), explaining 9.7 % of the variance in Bayley-III Cognitive Index scores (R^2^ = 0.097, adjusted R^2^ = 0.062). However, none of the individual predictors reached significance: *theta modulation index at 6 months* (standardized β = 0.16, 95 % CI [-0.21, 0.21], t = 1.458, p = 0.149), *absolute theta power at 6 months* (standardized β = 0.16, 95 % CI [-0.06, 0.38], t = 1.453, p = 0.150), and *age at outcome* (standardized β = 0.18, 95 % CI [-0.04, 0.39], t = 1.638, p = 0.106). These results suggest that theta activity indices at 6 months are not particularly predictive of later cognitive development.

Analogous analyses (including the 6-month and 12-month variables as separate predictors and repeated on larger samples) for the Visual attention and Language scores revealed that the best-fitting models did not generalize to larger samples (measures of theta activity as predictors had p > 0.14). Details of these analyses are reported in Supplementary Material (section S2). Additional analyses exploring the relation between adjusted change scores in theta activity indices from 6 months to 12 months and cognitive outcomes at 24 months revealed no significant effects (p > 0.06 in all three best-fitting models), these results are also reported in detail in Supplementary Material (section S3).

Specificity to the theta frequency band

In order to verify to which extent the investigated effects are specific to the theta frequency band, we performed analogous analyses using indices of alpha (6–9 Hz) activity. We found that similarly to theta, alpha power increased over the course of video viewing in 6-month-olds (t(97) = 8.08, p < 0.001) and 12-month-olds (t(86) = 2.91, p = 0.005).

Except for relative power, indices of alpha activity followed similar developmental trajectories as theta activity: an increase in absolute alpha power (t(67) = 13.44, p < .001), and decrease in alpha power modulation (t(67) = 2.09, p = 0.04) from 6 to 12 months. In contrast to the theta rhythm, relative alpha power increased in this time (t(67) = 11.08, p < 0.001).

Importantly, *composite absolute alpha power* turned out to be a significant positive predictor of the Bayley-III Cognitive index scores (standardized β = 0.33, 95 % CI [0.07, 0.59], t = 2.51, p = 0.014), while *composite relative alpha power* negatively predicted language outcomes (standardized β = −0.30, 95 % CI [-0.49, - 0.11], t = -3.11, p = 0.003) at 24 months.

The analyses involving indices of the alpha frequency band are reported in detail in Supplementary Material (section S4).

## 4 Discussion

Frontocentral theta oscillations have recently attracted a lot of scientific interest as a potential marker of cognitive development in preverbal infants (Braithwaite et al., 2020, Brandes-Aitken et al., 2023, Brandes-Aitken et al., 2023, Jones et al., 2020; Perone and Gartstein, 2019; Rico-Picó et al., 2025; Xie et al., 2018). The present study contributes to these broader efforts to improve our understanding of the role the theta rhythm plays in early cognition by examining developmental trajectories of three different indices of theta activity from 6 to 12 months: absolute theta power, relative theta power, and theta modulation index, and their predictive relation to cognitive level, selective attention, and language abilities at 24 months.

Firstly, we replicated the prior findings showing an increase in theta power over the course of viewing of a novel video (Braithwaite et al., 2020, Jones et al., 2020, Piccardi et al., 2020) in both 6- and 12-month-olds. Earlier research with adults showed theta power increases during working memory tasks and linked them to gradual synchronization of activity within cortico-hippocampal networks (see Sauseng et al., 2010 for a review). Our result further supports the notion that the theta rhythm plays a role in encoding of novel information already in early development (Begus and Bonawitz, 2020, Orekhova et al., 1999). Additionally, we found significant inter-individual variability in the indices of theta modulation by novel content at both ages. As previous research showed that this variability is predictive of later cognitive outcomes (Braithwaite et al., 2020, Jones et al., 2020), one of the aims of our study was to replicate this effect.

We used a composite score (of the 6- and 12-month-olds’ data) of the theta modulation index, together with composite scores of more established indices of theta activity, absolute and relative power, to predict cognitive outcomes at 24 months. Our analyses revealed that general cognitive level, as captured by the Bayley-III Cognitive Index, was best predicted by the composite score of absolute theta power at 6 and 12 months. In analyses including data from 6 and 12 months separately, we found some weak evidence for the pre-registered hypothesized link between the theta modulation index at 6 months and the Bayley-III Cognitive Index at 24 months, but this effect did not generalize to a larger sample size. Therefore, our results suggest that absolute power might be the most sensitive index of early cognitive development, at least when focusing on the frontocentral theta rhythm. Similarly, a recent study examining both absolute and relative frontocentral theta indices during non-social video viewing (a screensaver with moving abstract shapes) at 2 years as predictors of intelligence at 5 years found that absolute theta power, but not relative theta power, positively predicted later intelligence (Sullivan et al., 2025). As relative power indices are strongly affected by activity in other frequency bands (related or unrelated to the cognitive processes of interest), absolute theta indices may be better at capturing ‘pure’ task-related theta activity, particularly in contexts eliciting cognitive engagement (as in our study). Absolute EEG power, as compared with relative power, has also been previously found to be a more sensitive marker in some clinical contexts, such as characterizing encephalopathy severity (Govindan et al., 2017) or attention deficit hyperactivity disorder diagnostics (Markovska-Simoska and Pop-Jordanova, 2017).

The notion that absolute theta power might be a robust correlate of early cognition is further supported by our results pertaining to the intra-individual stability of the indices of theta activity. We found absolute theta power to be significantly correlated between 6 and 12 months, speaking for a degree of intra-individual stability suggesting a trait-like feature. While relative theta power showed a similar degree of intra-individual correlation between 6 and 12 months (as was previously found for relative alpha power, Marshall et al., 2002), the theta modulation index showed no such relation between the two ages. Additional analyses of intraclass correlation coefficients revealed values around 0.40 for absolute and relative theta power indices, but only 0.10 for the theta modulation index (in comparison, prior research reported that characteristics of event-related potentials collected one week apart in 9- to 10-month-olds were associated with ICC values ranging from 0.57 to 0.76; Munsters et al., 2019). In combination with the relatively low within-age reliability, our data suggests that the theta modulation index, while found to be a promising marker of early cognitive development in prior research (Braithwaite et al., 2020, Jones et al., 2020), may be outperformed by more established indices of theta activity. Of note, the previous studies reporting links between the theta modulation index and later cognition did not investigate the reliability of the measure or report analyses including absolute or relative theta power, therefore it remains unclear how these indices of theta activity would compare with the theta modulation index in the other cohorts. Importantly, although our data indicates absolute theta power as the most robust predictor of later cognitive development, experts in the field of infant EEG generally recommend reporting both absolute and relative power values in frequency-domain analyses (Troller-Renfree et al., 2025).

While we found that absolute theta power was predictive of later cognitive outcomes as measured with the Bayley-III Cognitive Index, none of the measures of theta activity were predictive of selective attention or language skills at 24 months, in our sample. Although this absence of evidence does not equal evidence for absence of an effect, our findings are consistent with previous studies that found frontocentral theta power indices in the first two years of life to be predictive of later general cognitive abilities (Jones et al., 2020, Sullivan et al., 2025, Tan et al., 2023) but not of specific cognitive skills (Sullivan et al., 2025). Additionally, theta indices measured at 6 or 12 months were not particularly robust predictors of later cognition. Instead, composite scores of theta activity aggregated across multiple measurements demonstrated the strongest predictive properties, consistent with findings by Tan et al. (2023). Overall, it appears that theta activity might index general cognitive processing abilities, with relatively stable individual differences in early development.

Notably, in an additional analysis, we found that a number of effects of interest were not specific to the theta rhythm, but could also be found in the alpha frequency band. These effects included the increase in power over the course of video viewing, and the predictive relationship between absolute power and later cognitive outcomes. Considering that a number of studies on the functionality of the theta rhythm in development did not report analyses on other frequency bands (e.g. Braithwaite et al., 2020; Jones et al., 2020), our finding calls into question the specificity of some of the previously reported effects to the theta band. Indeed, a study reporting task engagement-dependent changes in frontocentral theta power in 4-year-olds found similar changes in the alpha band (Meyer et al., 2019). Additionally, although one study reported a longitudinal predictive relationship between EEG power in early childhood and later cognitive outcomes specific to the theta frequency band (Tan et al., 2023), another found that EEG power in both theta and alpha frequency bands in infancy was a positive predictor of later cognitive outcomes (consistent with our own findings; Rico-Picó et al., 2025). Frontal alpha power at 5 months was also recently reported to differ between infants who will and will not later receive an attention deficit hyperactivity disorder diagnosis, indicating early differences in information processing (Eng et al., 2025). Furthermore, there is evidence that EEG activity in higher frequency bands (> 24 Hz) recorded at birth is predictive of later cognition (Brito et al., 2016). Future research should carefully investigate claims about the functional specificity of the theta rhythm by incorporating other frequency bands in the analyses.

Our investigation into the group-level trajectories of fronto-medial theta activity development from 6 to 12 months revealed patterns consistent with prior reports: an increase in absolute theta power (Gabard-Durnam et al., 2019, Jing et al., 2010, Rico-Picó et al., 2023, Wilkinson et al., 2023, Wilkinson et al., 2024), and a decrease in relative power (Rico-Picó et al., 2023). Previously not investigated in terms of its longitudinal development, the theta modulation index showed a decrease from 6 to 12 months, but also a large interindividual variability in change trajectories. The observed patterns of change in the EEG power spectrum from 6 to 12 months have been previously linked to synaptogenesis, as well as maturation of the inhibitory network and thalamocortical circuits (Antón-Bolaños et al., 2018, Wilkinson et al., 2023, Wilkinson et al., 2024). The diverging trajectories of absolute and relative theta band power development might reflect parallel developmental processes: the maturation of theta oscillatory activity as well as broadband neuronal firing in the former case, and the relatively faster increases in oscillatory power in higher frequency bands in the latter case (Marshall et al., 2002, Wilkinson et al., 2023). Of note, some recent relevant studies (Rico-Picó et al., 2023, Rico-Picó et al., 2025) used analyses approaches allowing for the separation of aperiodic and periodic activity contributions to the power spectrum (Donoghue et al., 2020, Schaworonkow and Voytek, 2021), demonstrating divergent association patterns with measures of executive control in infancy and toddlerhood. Future research employing this kind of analysis should closely examine the relationships between these two aspects of EEG activity and different aspects of cognitive development, as such separation would help disentangle contributions of truly oscillatory changes from broadband shifts in neural activity. Additionally, prospective studies may further employ machine learning approaches to predict developmental outcomes from infant EEG data (Ng et al., 2022, Scheinost et al., 2023).

Although theta activity has received growing attention in recent years as a potential neural marker for early cognitive development, methodological uncertainties remain regarding the selection of appropriate theta power indices. By examining absolute power, relative power and theta modulation at 6 and 12 months of age, and their predictive value for cognition, attention and language at 24 months, this longitudinal study provides new insights into the reliability, stability and predictive potential of these commonly used theta power indices. Our findings point to absolute theta power as the most sensitive predictor of later cognitive outcomes, making it a promising neural marker for future research on the role of theta rhythm in early cognitive development. However, we found that the link to cognitive development was not limited to the theta band but could be found for the alpha band as well, underscoring the importance of incorporating activity from other EEG frequency bands in future research. As interest in identifying early predictors of cognitive development continues to grow, our study offers both methodological and empirical insights to guide future efforts in this domain.

## CRediT authorship contribution statement

**Johanna Ruess:** Writing – review & editing, Writing – original draft, Investigation, Formal analysis. **Stefanie Hoehl:** Writing – review & editing, Supervision, Resources, Methodology, Funding acquisition, Conceptualization. **Martina Arioli:** Writing – review & editing, Validation. **Regina Ori Stoeckl:** Writing – review & editing, Writing – original draft, Project administration, Investigation. **Alicja Brzozowska:** Writing – review & editing, Writing – original draft, Visualization, Project administration, Methodology, Investigation, Formal analysis, Data curation, Conceptualization.

## Declaration of Competing Interest

The authors declare that they have no known competing financial interests or personal relationships that could have appeared to influence the work reported in this paper.

## Data Availability

The analysis scripts and data supporting the results of the study are openly available at https://github.com/alicja444/ThetaActivity and https://osf.io/63ena/, respectively.
