# Supplementary material for: Theta activity as a marker of cognitive development in infancy: A longitudinal study across the first two years of life
Source: Dev Cogn Neurosci. 2025 Nov 4;76:101642. doi: 10.1016/j.dcn.2025.101642 (PMC12793793; doi:10.1016/j.dcn.2025.101642)
Supplement: Supplementary file 1 — Supplementary material [file mmc1.docx]

**Theta activity as a marker of cognitive development in infancy: a longitudinal study across the first two years of life**

Supplementary material

S1) Reasons for missing data

S2) Analyses of Visual attention & Language analyses with larger samples

S3) Analyses including adjusted change scores

S4) Analyses with the alpha band

S5) The impact of total absolute power range on the relative power indices

S6) The impact of relative theta calculation method on the investigated effects

S7) Associations of outcome variables with demographic variables

S8) Exploring correlations between indices of theta activity

S9) Steps in the selection of the best-fitting models in predicting cognitive outcomes

S10) Examining the linear assumptions of the theta modulation index

S1) Reasons for missing data

Table S1. Reasons for missing data

| Timepoint | Number of infants participating in the study | Measure | Number of valid datasets | Reasons for missingness |
| --- | --- | --- | --- | --- |
| T1 (6 months) | 140 | EEG data | 98 | no EEG collected due to fussiness (5); experimenter error (3); technical problems during data acquisition (8); cross-correlation value across all frequencies before and after wavelet thresholding below 0.1 (4); fewer than 10 good quality segments (22) |
| T2 (12 months) | 133 | EEG data | 87 | no EEG collected due to fussiness (11); experimenter error (1); technical problems during data acquisition (1); cross-correlation value across all frequencies before and after wavelet thresholding below 0.1 (2); fewer than 10 good quality segments (31) |
| T3 (24 months) | 122 | Bayley-III Cognitive Index | 117 | child did not want to participate (2); experimenter error (3) |
|  |  | Selective Attention score | 111 | child had a score of 0 which should be treated as ‘missing’ according to the task manual (5); child did not want to participate (6) |
|  |  | ELFRA-2 (Language) | 115 | parent did not fill out the questionnaire (7) |

S2) Predicting Selective attention and Language with 6 month and 12 month theta activity indices as separate predictors

The full model for each outcome variable included the following predictors: *age at outcome*, *absolute theta power at 6 months*, *absolute theta power at 12 months*, *relative theta power at 6 months*, *relative theta power at 12 months*, *theta modulation index at 6 months*, and *theta modulation index at 12 months*.

1. Visual attention

The best-fitting model predicting Visual attention included only one predictor, *theta modulation index at 6 months* (F(1, 52) = 3.49) and was marginally non-significant (p = 0.067), with R^2^ = 0.063 and adjusted R^2^ = 0.045. *Theta modulation index at 6 months* was a marginally non-significant positive predictor of Selective attention at 24 months (standardized β = 0.25, 95% CI [-0.02, 0.52], p = 0.067). Since this model only included a predictor variable from the 6 month timepoint, we fitted the model again, on a sample of participants who had 6 month (but not necessarily 12 month) data available, to verify the model in this larger sample.

In the larger sample, the model was not significant (F(1, 73) = 0.001, p = 0.94), speaking against the predictive value of theta modulation index at 6 months for visual attention at 24 months.

1. Language

The best-fitting model predicting Language included *absolute theta power at 12 months* and *age at outcome* as predictors (F(2, 52) and was marginally non-significant (p = 0.067). Neither *absolute theta power at 12 months* (standardized β = -0.21, 95% CI [-0.47, 0.05], p = 0.117) nor *age at outcome* (standardized β = 0.24, 95% CI [-0.02, 0.51], p = 0.071) were significant predictors of Language. Because this model only included a predictor variable from the 12 month timepoint, we fitted the model again, on a sample of participants who had 12 month (but not necessarily 6 month) data available, to verify the model in this larger sample.

In the larger sample, the model was significant (F(2, 70) = 4.17, p = 0.012; R^2^ = 0.106, adjusted R^2^ = 0.081). However, only *age at outcome* (standardized β = 0.28, 95% CI [0.05, 0.50], p = 0.016) but not *absolute theta power at 12 months* (standardized β = -0.17, 95% CI [-0.39, 0.06], p = 0.139) was a significant predictor of Language at 24 months. This result speaks against the predictive value of absolute theta power at 12 months for language at 24 months.

S3) Analyses including adjusted change scores

In order to explore our longitudinal data in more depth, in addition to analyses including composite scores of indices of theta activity as well as indices of theta activity at individual time points, we also conducted an analysis focusing on adjusted change scores of indices of theta activity. The adjusted change scores were calculated by regressing the change in values between 6 and 12 months of a given index of theta activity (absolute power, relative power, and theta modulation) on the value at 6 months and extracting the residuals. These scores therefore captured the change in theta activity from 6 to 12 months, while controlling for values at baseline (6 months). Because of divergent patterns of group-level changes in indices of theta activity (increase in absolute power, but decrease in relative power and theta modulation index from 6 to 12 months), values at 6 months were subtracted from values at 12 months for absolute theta power, but values at 12 months were subtracted from values at 6 months for relative theta power and theta modulation index. Thus, positive values of adjusted change scores index changes in the normative (consistent with group-level developmental patterns) direction, while negative values index changes against the normative direction.

The full model for each outcome variable included the following predictors: *adjusted change score of absolute theta power*, *adjusted change score of relative theta power*, *adjusted change score of theta modulation index* and *age at outcome*.

1. Bayley-III Cognitive Index

The best-fitting model predicting the Bayley-III Cognitive Index included only age at outcome as a predictor (standardized β = 0.18, 95% CI [-0.08, 0.45], p = 0.161) and was not significant (F(1, 57) = 2.02, p = 0.161).

1. Visual attention

The best-fitting model predicting Visual attention included only adjusted change score of absolute theta power as a predictor (standardized β = 0.20, 95% CI [-0.08, 0.47], p = 0.154) and was not significant (F(1, 52) = 2.09, p = 0.154).

1. Language

The best-fitting model predicting Language was significant (F(2, 52) = 3.39, p = 0.041) and included two marginally non-significant predictors: adjusted change score of absolute theta power (standardized β = - 0.25, 95% CI [-0.51, 0.02], p = 0.065) and age at outcome (standardized β = 0.25, 95% CI [-0.02, 0.51], p = 0.065), explaining 11,6% of variance in Language (R^2^ = 0.116, adjusted R^2^ = 0.081). The direction of the relation suggests that larger increases in absolute theta power from 6 to 12 months may be associated with more negative Language outcomes at 24 months, but the result is not significant so should be interpreted with caution.

S4) Analyses including the alpha frequency band

1. Correlations between indices of alpha activity between 6 and 12 months

**Absolute alpha power** was highly correlated between 6 and 12 months (r(67) = 0.60, p < .001).

**Relative alpha power** was also highly correlated between 6 and 12 months (r(67) = 0.60, p < .001).

**Alpha power modulation** was not correlated between 6 and 12 months (r(67) = -0.07, p = .587).

1. Predicting cognitive development with indices of alpha activity

**Bayley scales at 24 months**

The best-fitting model included composite absolute alpha power, composite relative alpha power and age at outcome as predictors (F(3, 92) = 3.61, p = .016), explaining 10.5% of the variance in the outcome variable (R^2^ = 0.105, adjusted R^2^ = 0.076). The only significant predictor in the model was the composite absolute alpha power (standardized β = 0.33, 95% CI [0.07, 0.59], t =2.51, p = 0.014). Composite relative alpha power (standardized β = -0.19, 95% CI [- 0.45, 0.07], t = - 1.45, p =0.15) and age at outcome (standardized β = 0.19, 95% CI [-0.01, 0.39], t = 1.93, p = 0.057) did not reach significance as predictors.

**Visual attention**

The best-fitting model was a null model.

**Language**

The best-fitting model included two predictors: composite relative alpha power and age at outcome (F(2, 91) = 8.68, p < .001), explaining 16% of the variance in the outcome variable (R^2^ = 0.160, adjusted R^2^ = 0.142). Composite relative alpha power was a negative predictor of language outcomes (standardized β = -0.30, 95% CI [-0.49, - 0.11], t = -3.11, p = 0.003), while age at outcome predicted language positively (standardized β = 0.25, 95% CI [0.06, 0.45], t =2.65, p = 0.009).

S5) The impact of total absolute power range on the relative power indices

The calculation of relative power indices requires the calculation of power within the frequency band of interest, divided by total absolute power. As the definitions of “total absolute power” vary, here we briefly examine whether our choice to calculate total absolute power within the frequency range of 1 to 45 Hz (subsequently referred to as approach A) had a significant impact on our results by comparing it with an approach employed by Rico‐Picó et al. (2023), who chose 1-20 Hz as their range for total power (subsequently referred to as approach B).

1. Association between relative theta power indices calculated with approach A and approach B at 6 and 12 months

At both 6 months (r(96) = 0.88, p < .001) and 12 months (r(85) = 0.93, p < .001) the two relative theta indices were highly correlated.

1. Developmental trajectory of relative theta power (approach B)

Similarly to relative power calculated with approach A (see main manuscript), relative power calculated with approach B decreased from 6 to 12 months (t(67) = -10.06, p < .001).

1. Predicting cognitive outcomes at 24 months with relative theta calculated with approach B:

We repeated the analyses described in the main manuscript, this time with relative theta index calculated with approach B.

Bayley-III Cognitive index:

The best model was identical to the one chosen when relative theta power calculated with approach A was used (the best model did not include composite relative theta power).

Selective attention:

The best model was identical to the one chosen when relative theta power calculated with approach A was used (null model).

Language:

The best model was identical to the one chosen when relative theta power calculated with approach A was used (only age at outcome was a significant predictor)

In conclusion, the choice of frequency range for total absolute power calculation did not have a significant impact on our results.

S6) The impact of relative theta calculation method on the investigated effects

In our study, we calculated relative power indices by averaging absolute power in the theta frequency band across the electrodes of interest and then dividing the value by total absolute power averaged across the electrodes on interest (treating all electrodes as one “region of interest”; subsequently referred to as approach R). However, relative power can also be calculated by first computing the ratio of theta power to absolute power per electrode, and then averaging across electrodes, which might lead to slightly different results. In order to check whether this analytical choice had a significant impact on our findings, we rerun our analyses, this time including relative power indices calculated with the alternative approach (subsequently referred to as approach E).

1. Association between relative theta power indices calculated with approach R and approach E at 6 and 12 months

The relative power values calculated with the two approaches were very highly correlated at both 6 months (r(96) = 0.97, p < .001) and 12 months (r(85) = 0.98, p < .001).

1. Developmental trajectory of relative theta power (approach E)

Similarly to relative power calculated with approach A (see main manuscript), relative power calculated with approach E decreased from 6 to 12 months (t(67) = -8.10, p < .001).

1. Predicting cognitive outcomes at 24 months with relative theta calculated with approach E:

We repeated the analyses described in the main manuscript, this time with relative theta index calculated with approach E.

Bayley-III Cognitive index:

The best model was identical to the one chosen when relative theta power calculated with approach R was used (the best model did not include composite relative theta power).

Selective attention:

The best model was identical to the one chosen when relative theta power calculated with approach R was used (null model).

Language:

The best model was identical to the one chosen when relative theta power calculated with approach R was used (only age at outcome was a significant predictor)

In conclusion, our analytical approach to calculating relative theta power did not have a significant impact on our results, as compared with the alternative approach.

S7) Associations of outcome variables with demographic variables

1. Sex

| Independent Samples T-Test (positive values indicate higher scores in female infants) | | | | | | | |
| --- | --- | --- | --- | --- | --- | --- | --- |
|  | | **t** | | **df** | | **p** | |
| Bayley-III Cognitive Index |  | 0.716 |  | 115 |  | 0.475 |  |
| Selective attention |  | 1.912 |  | 109 |  | 0.058 |  |
| ELFRA_Production |  | 0.772 |  | 113 |  | 0.442 |  |
| ELFRA_Syntax |  | 1.722 |  | 113 |  | 0.088 |  |
| ELFRA_Morphology |  | 1.401 |  | 113 |  | 0.164 |  |
|  | | | | | | | |
| *Note.*  Student's t-test. | | | | | | | |

1. Gestational age

| **Variable** | |  | | **Gestational age** | |  |  |  |  |
| --- | --- | --- | --- | --- | --- | --- | --- | --- | --- |
| Bayley-III Cognitive Index |  | Pearson's r |  | 0.189 |  |  |  |  |  |
|  |  | p-value |  | 0.042 |  |  |  |  |  |
| Selective attention |  | Pearson's r |  | 0.014 |  |  |  |  |  |
|  |  | p-value |  | 0.883 |  |  |  |  |  |
| ELFRA_Production |  | Pearson's r |  | 0.107 |  |  |  |  |  |
|  |  | p-value |  | 0.260 |  |  |  |  |  |
| ELFRA_Syntax |  | Pearson's r |  | 0.133 |  |  |  |  |  |
|  |  | p-value |  | 0.161 |  |  |  |  |  |
| ELFRA_Morphology |  | Pearson's r |  | 0.048 |  |  |  |  |  |
|  |  | p-value |  | 0.611 |  |  |  |  |  |

1. Household income

Household income was an ordinal variable with the following values: 1 – below 26 000€ annually, 2 – between 26 000 and 45 000€ annually and 3 – more than 45 000 annually.

| ANOVA – Bayley-III Cognitive Index | | | | | | | | | | | |
| --- | --- | --- | --- | --- | --- | --- | --- | --- | --- | --- | --- |
| **Cases** | | **Sum of Squares** | | **df** | | **Mean Square** | | **F** | | **p** | |
| household_income |  | 78.281 |  | 2 |  | 39.141 |  | 1.797 |  | 0.171 |  |
| Residuals |  | 2221.052 |  | 102 |  | 21.775 |  |  |  |  |  |
|  | | | | | | | | | | | |
| *Note.*  Type III Sum of Squares | | | | | | | | | | | |

| ANOVA – Selective attention | | | | | | | | | | | |
| --- | --- | --- | --- | --- | --- | --- | --- | --- | --- | --- | --- |
| **Cases** | | **Sum of Squares** | | **df** | | **Mean Square** | | **F** | | **p** | |
| household_income |  | 5.364 |  | 2 |  | 2.682 |  | 1.448 |  | 0.240 |  |
| Residuals |  | 177.817 |  | 96 |  | 1.852 |  |  |  |  |  |
|  | | | | | | | | | | | |
| *Note.*  Type III Sum of Squares | | | | | | | | | | | |

| ANOVA - ELFRA_Production | | | | | | | | | | | |
| --- | --- | --- | --- | --- | --- | --- | --- | --- | --- | --- | --- |
| **Cases** | | **Sum of Squares** | | **df** | | **Mean Square** | | **F** | | **p** | |
| household_income |  | 2858.092 |  | 2 |  | 1429.046 |  | 0.416 |  | 0.661 |  |
| Residuals |  | 346701.293 |  | 101 |  | 3432.686 |  |  |  |  |  |
|  | | | | | | | | | | | |
| *Note.*  Type III Sum of Squares | | | | | | | | | | | |

| ANOVA - ELFRA_Syntax | | | | | | | | | | | |
| --- | --- | --- | --- | --- | --- | --- | --- | --- | --- | --- | --- |
| **Cases** | | **Sum of Squares** | | **df** | | **Mean Square** | | **F** | | **p** | |
| household_income |  | 253.759 |  | 2 |  | 126.880 |  | 0.811 |  | 0.447 |  |
| Residuals |  | 15796.702 |  | 101 |  | 156.403 |  |  |  |  |  |
|  | | | | | | | | | | | |
| *Note.*  Type III Sum of Squares | | | | | | | | | | | |

| ANOVA - Elfra_morph | | | | | | | | | | | |
| --- | --- | --- | --- | --- | --- | --- | --- | --- | --- | --- | --- |
| **Cases** | | **Sum of Squares** | | **df** | | **Mean Square** | | **F** | | **p** | |
| household_income |  | 4.553 |  | 2 |  | 2.277 |  | 0.119 |  | 0.888 |  |
| Residuals |  | 1935.293 |  | 101 |  | 19.161 |  |  |  |  |  |
|  | | | | | | | | | | | |
| *Note.*  Type III Sum of Squares | | | | | | | | | | | |

S8) Exploring correlations between indices of theta activity

The table below shows correlations between indices of theta activity.

| **Pearson's Correlations** | | | | | | | | | | | | | | | | | | | |  |
| --- | --- | --- | --- | --- | --- | --- | --- | --- | --- | --- | --- | --- | --- | --- | --- | --- | --- | --- | --- | --- |
| **Variable** | |  | **theta_modulationT1** | | | **theta_modulationT2** | | **absolute_thetaT1** | | | **absolute_thetaT2** | | | **relative_thetaT1** | | | **relative_thetaT2** | | |  |
| 1. theta_modulationT1 |  | Pearson's r |  | — |  |  |  | |  |  | |  |  | |  |  | |  |  | |
|  |  | p-value |  | — |  |  |  | |  |  | |  |  | |  |  | |  |  | |
| 2. theta_modulationT2 |  | Pearson's r |  | 0.094 |  | — |  | |  |  | |  |  | |  |  | |  |  | |
|  |  | p-value |  | 0.445 |  | — |  | |  |  | |  |  | |  |  | |  |  | |
| 3. absolute_thetaT1 |  | Pearson's r |  | 0.031 |  | -0.084 |  | | — |  | |  |  | |  |  | |  |  | |
|  |  | p-value |  | 0.763 |  | 0.495 |  | | — |  | |  |  | |  |  | |  |  | |
| 4. absolute_thetaT2 |  | Pearson's r |  | 0.133 |  | -0.063 |  | | 0.402 |  | | — |  | |  |  | |  |  | |
|  |  | p-value |  | 0.279 |  | 0.565 |  | | < .001 |  | | — |  | |  |  | |  |  | |
| 5. relative_thetaT1 |  | Pearson's r |  | -0.094 |  | -0.048 |  | | 0.082 |  | | 0.148 |  | | — |  | |  |  | |
|  |  | p-value |  | 0.359 |  | 0.699 |  | | 0.425 |  | | 0.228 |  | | — |  | |  |  | |
| 6. relative_thetaT2 |  | Pearson's r |  | -0.094 |  | -0.154 |  | | 0.042 |  | | 0.186 |  | | 0.351 |  | | — |  | |
|  |  | p-value |  | 0.447 |  | 0.153 |  | | 0.734 |  | | 0.084 |  | | 0.003 |  | | — |  | |
|  | | | | | | | | | | | | | | | | | | | |  |

S9) Steps in the selection of the best-fitting models in predicting cognitive outcomes

In predicting cognitive outcomes at 24 months, we used the stepAIC() function in the R package MASS (Venables & Ripley, 2002) to select the linear regression models that had the best fit. Below, we provide details of the models considered (and rejected) in the stepwise selection process.

1. Predicting Bayley-III with theta absolute (absthetaz), relative (relthetaz) and modulation (thetamodulz) composite scores, controlling for age at outcome:

Model 1 Summary:

Call:

lm(formula = Bayley ~ absthetaz + thetamodulz + relthetaz + Age_at_T3,

data = DB_clean_Bayley)

Residuals:

Min 1Q Median 3Q Max

-11.0218 -2.8296 -0.2755 3.1482 9.2284

Coefficients:

Estimate Std. Error t value Pr(>|t|)

(Intercept) 31.93191 18.74053 1.704 0.0918 .

absthetaz 1.07681 0.50275 2.142 0.0349 *

thetamodulz -0.05782 0.53549 -0.108 0.9142

relthetaz -0.39202 0.47305 -0.829 0.4094

Age_at_T3 0.04768 0.02519 1.893 0.0616 .

---

Signif. codes: 0 ‘***’ 0.001 ‘**’ 0.01 ‘*’ 0.05 ‘.’ 0.1 ‘ ’ 1

Residual standard error: 4.406 on 91 degrees of freedom

Multiple R-squared: 0.0932, Adjusted R-squared: 0.05334

F-statistic: 2.338 on 4 and 91 DF, p-value: 0.06111

AIC = 289.6

Model 2 Summary:

Call:

lm(formula = Bayley ~ absthetaz + relthetaz + Age_at_T3, data = DB_clean_Bayley)

Residuals:

Min 1Q Median 3Q Max

-10.9735 -2.8084 -0.2489 3.1271 9.1871

Coefficients:

Estimate Std. Error t value Pr(>|t|)

(Intercept) 31.95956 18.63785 1.715 0.0898 .

absthetaz 1.06706 0.49190 2.169 0.0326 *

relthetaz -0.38307 0.46322 -0.827 0.4104

Age_at_T3 0.04764 0.02505 1.901 0.0604 .

---

Signif. codes: 0 ‘***’ 0.001 ‘**’ 0.01 ‘*’ 0.05 ‘.’ 0.1 ‘ ’ 1

Residual standard error: 4.382 on 92 degrees of freedom

Multiple R-squared: 0.09309, Adjusted R-squared: 0.06351

F-statistic: 3.148 on 3 and 92 DF, p-value: 0.0288

AIC = 287.61

Model 3 was the best-fitting model (described in the manuscript).

1. Predicting selective attention scores with theta absolute (absthetaz), relative (relthetaz) and modulation (thetamodulz) composite scores, controlling for age at outcome:

Model 1 Summary:

Call:

lm(formula = Visual_search ~ absthetaz + thetamodulz + relthetaz +

Age_at_T3, data = DB_clean_VSearch)

Residuals:

Min 1Q Median 3Q Max

-2.94805 -0.84478 -0.08587 0.88773 2.85668

Coefficients:

Estimate Std. Error t value Pr(>|t|)

(Intercept) 0.644945 5.479137 0.118 0.907

absthetaz -0.110297 0.149796 -0.736 0.464

thetamodulz -0.120162 0.165103 -0.728 0.469

relthetaz 0.169106 0.148195 1.141 0.257

Age_at_T3 0.003803 0.007356 0.517 0.606

Residual standard error: 1.31 on 86 degrees of freedom

Multiple R-squared: 0.0312, Adjusted R-squared: -0.01386

F-statistic: 0.6923 on 4 and 86 DF, p-value: 0.5993

AIC = 53.98

Model 2 Summary:

Call:

lm(formula = Visual_search ~ absthetaz + thetamodulz + relthetaz,

data = DB_clean_VSearch)

Residuals:

Min 1Q Median 3Q Max

-3.01208 -0.89079 -0.09178 0.87986 2.86226

Coefficients:

Estimate Std. Error t value Pr(>|t|)

(Intercept) 3.4770 0.1370 25.386 <2e-16 ***

absthetaz -0.1056 0.1489 -0.709 0.480

thetamodulz -0.1147 0.1641 -0.699 0.486

relthetaz 0.1605 0.1466 1.094 0.277

---

Signif. codes: 0 ‘***’ 0.001 ‘**’ 0.01 ‘*’ 0.05 ‘.’ 0.1 ‘ ’ 1

Residual standard error: 1.304 on 87 degrees of freedom

Multiple R-squared: 0.02818, Adjusted R-squared: -0.005326

F-statistic: 0.8411 on 3 and 87 DF, p-value: 0.475

AIC = 52.26

Model 3 Summary:

Call:

lm(formula = Visual_search ~ absthetaz + relthetaz, data = DB_clean_VSearch)

Residuals:

Min 1Q Median 3Q Max

-3.11379 -0.88079 -0.06318 0.84282 2.99448

Coefficients:

Estimate Std. Error t value Pr(>|t|)

(Intercept) 3.4761 0.1366 25.455 <2e-16 ***

absthetaz -0.1228 0.1464 -0.839 0.404

relthetaz 0.1780 0.1440 1.236 0.220

---

Signif. codes: 0 ‘***’ 0.001 ‘**’ 0.01 ‘*’ 0.05 ‘.’ 0.1 ‘ ’ 1

Residual standard error: 1.301 on 88 degrees of freedom

Multiple R-squared: 0.02272, Adjusted R-squared: 0.0005105

F-statistic: 1.023 on 2 and 88 DF, p-value: 0.3638

AIC = 50.77

Model 4 Summary:

Call:

lm(formula = Visual_search ~ relthetaz, data = DB_clean_VSearch)

Residuals:

Min 1Q Median 3Q Max

-3.15750 -0.81304 -0.00718 0.83554 3.11705

Coefficients:

Estimate Std. Error t value Pr(>|t|)

(Intercept) 3.4812 0.1362 25.561 <2e-16 ***

relthetaz 0.1661 0.1431 1.161 0.249

---

Signif. codes: 0 ‘***’ 0.001 ‘**’ 0.01 ‘*’ 0.05 ‘.’ 0.1 ‘ ’ 1

Residual standard error: 1.298 on 89 degrees of freedom

Multiple R-squared: 0.01491, Adjusted R-squared: 0.003844

F-statistic: 1.347 on 1 and 89 DF, p-value: 0.2488

AIC = 49.5

Model 5 (null model) was the best-fitting model (described in the manuscript).

1. Language scores with theta absolute (absthetaz), relative (relthetaz) and modulation (thetamodulz) composite scores, controlling for age at outcome:

Model 1 Summary:

Call:

lm(formula = Language ~ absthetaz + thetamodulz + relthetaz +

Age_at_T3, data = DB_clean_Language)

Residuals:

Min 1Q Median 3Q Max

-2.0632 -0.4922 0.1153 0.6541 2.0578

Coefficients:

Estimate Std. Error t value Pr(>|t|)

(Intercept) -10.421677 3.911717 -2.664 0.00916 **

absthetaz -0.036089 0.108289 -0.333 0.73972

thetamodulz -0.126083 0.113276 -1.113 0.26868

relthetaz -0.022808 0.101891 -0.224 0.82339

Age_at_T3 0.013993 0.005254 2.664 0.00918 **

---

Signif. codes: 0 ‘***’ 0.001 ‘**’ 0.01 ‘*’ 0.05 ‘.’ 0.1 ‘ ’ 1

Residual standard error: 0.9325 on 89 degrees of freedom

Multiple R-squared: 0.0868, Adjusted R-squared: 0.04576

F-statistic: 2.115 on 4 and 89 DF, p-value: 0.08552

AIC = -8.28

Model 2 Summary:

Call:

lm(formula = Language ~ absthetaz + thetamodulz + Age_at_T3,

data = DB_clean_Language)

Residuals:

Min 1Q Median 3Q Max

-2.09575 -0.46777 0.09705 0.66456 2.05178

Coefficients:

Estimate Std. Error t value Pr(>|t|)

(Intercept) -10.512089 3.870221 -2.716 0.00792 **

absthetaz -0.039265 0.106788 -0.368 0.71397

thetamodulz -0.122702 0.111670 -1.099 0.27479

Age_at_T3 0.014115 0.005198 2.715 0.00793 **

---

Signif. codes: 0 ‘***’ 0.001 ‘**’ 0.01 ‘*’ 0.05 ‘.’ 0.1 ‘ ’ 1

Residual standard error: 0.9276 on 90 degrees of freedom

Multiple R-squared: 0.08628, Adjusted R-squared: 0.05583

F-statistic: 2.833 on 3 and 90 DF, p-value: 0.04271

AIC = -10.22

Model 3 Summary:

Call:

lm(formula = Language ~ thetamodulz + Age_at_T3, data = DB_clean_Language)

Residuals:

Min 1Q Median 3Q Max

-2.0949 -0.5099 0.1269 0.6930 2.0462

Coefficients:

Estimate Std. Error t value Pr(>|t|)

(Intercept) -10.476777 3.850601 -2.721 0.00780 **

thetamodulz -0.128891 0.109869 -1.173 0.24380

Age_at_T3 0.014069 0.005172 2.720 0.00781 **

---

Signif. codes: 0 ‘***’ 0.001 ‘**’ 0.01 ‘*’ 0.05 ‘.’ 0.1 ‘ ’ 1

Residual standard error: 0.9231 on 91 degrees of freedom

Multiple R-squared: 0.08491, Adjusted R-squared: 0.0648

F-statistic: 4.222 on 2 and 91 DF, p-value: 0.01764

AIC = -12.08

Model 4 was the best-fitting model (described in the manuscript).

1. Predicting Bayley-III Cognitive Index with 6- and 12-month variables as separate predictors, and controlling for age at outcome: theta modulation at 6 (modulation_indexT1theta) and 12 (modulation_indexT2theta) months, absolute theta power at 6 (absolute_powerT1theta) and 12 (absolute_powerT2theta) months, relative theta power at 6 (relative_powerT1theta) and 12 (relative_powerT2theta) months.

Model 1 Summary:

Call:

lm(formula = Bayley ~ modulation_indexT1theta + modulation_indexT2theta +

absolute_powerT1theta + absolute_powerT2theta + relative_powerT1theta +

relative_powerT2theta + Age_at_T3, data = DB_clean_Bayley2)

Residuals:

Min 1Q Median 3Q Max

-9.6576 -3.1309 0.1155 3.0095 8.3407

Coefficients:

Estimate Std. Error t value Pr(>|t|)

(Intercept) 28.47912 24.32957 1.171 0.2472

modulation_indexT1theta 6.52646 3.54900 1.839 0.0717 .

modulation_indexT2theta -2.64767 2.69910 -0.981 0.3312

absolute_powerT1theta 0.85333 0.64239 1.328 0.1900

absolute_powerT2theta 0.05102 0.69709 0.073 0.9419

relative_powerT1theta -4.92850 15.49671 -0.318 0.7518

relative_powerT2theta -2.67859 14.94350 -0.179 0.8585

Age_at_T3 0.04480 0.03103 1.444 0.1549

---

Signif. codes: 0 ‘***’ 0.001 ‘**’ 0.01 ‘*’ 0.05 ‘.’ 0.1 ‘ ’ 1

Residual standard error: 4.553 on 51 degrees of freedom

Multiple R-squared: 0.1562, Adjusted R-squared: 0.04044

F-statistic: 1.349 on 7 and 51 DF, p-value: 0.2471

AIC = 186.28

Model 2 Summary:

Call:

lm(formula = Bayley ~ modulation_indexT1theta + modulation_indexT2theta +

absolute_powerT1theta + relative_powerT1theta + relative_powerT2theta +

Age_at_T3, data = DB_clean_Bayley2)

Residuals:

Min 1Q Median 3Q Max

-9.6340 -3.1456 0.1417 2.9699 8.3771

Coefficients:

Estimate Std. Error t value Pr(>|t|)

(Intercept) 28.54027 24.08155 1.185 0.2413

modulation_indexT1theta 6.59847 3.37711 1.954 0.0561 .

modulation_indexT2theta -2.66109 2.66699 -0.998 0.3230

absolute_powerT1theta 0.87058 0.59185 1.471 0.1473

relative_powerT1theta -4.88605 15.33702 -0.319 0.7513

relative_powerT2theta -2.42217 14.38737 -0.168 0.8670

Age_at_T3 0.04496 0.03066 1.466 0.1486

---

Signif. codes: 0 ‘***’ 0.001 ‘**’ 0.01 ‘*’ 0.05 ‘.’ 0.1 ‘ ’ 1

Residual standard error: 4.51 on 52 degrees of freedom

Multiple R-squared: 0.1562, Adjusted R-squared: 0.05879

F-statistic: 1.604 on 6 and 52 DF, p-value: 0.1648

AIC = 184.28

Model 3 Summary:

Call:

lm(formula = Bayley ~ modulation_indexT1theta + modulation_indexT2theta +

absolute_powerT1theta + relative_powerT1theta + Age_at_T3,

data = DB_clean_Bayley2)

Residuals:

Min 1Q Median 3Q Max

-9.5784 -3.0758 0.1705 2.9309 8.4624

Coefficients:

Estimate Std. Error t value Pr(>|t|)

(Intercept) 28.24464 23.79626 1.187 0.2405

modulation_indexT1theta 6.57026 3.34189 1.966 0.0545 .

modulation_indexT2theta -2.62813 2.63530 -0.997 0.3232

absolute_powerT1theta 0.88000 0.58378 1.507 0.1376

relative_powerT1theta -5.98840 13.74134 -0.436 0.6648

Age_at_T3 0.04450 0.03026 1.471 0.1473

---

Signif. codes: 0 ‘***’ 0.001 ‘**’ 0.01 ‘*’ 0.05 ‘.’ 0.1 ‘ ’ 1

Residual standard error: 4.468 on 53 degrees of freedom

Multiple R-squared: 0.1557, Adjusted R-squared: 0.07605

F-statistic: 1.955 on 5 and 53 DF, p-value: 0.1007

AIC = 182.31

Model 4 Summary:

Call:

lm(formula = Bayley ~ modulation_indexT1theta + modulation_indexT2theta +

absolute_powerT1theta + Age_at_T3, data = DB_clean_Bayley2)

Residuals:

Min 1Q Median 3Q Max

-9.938 -2.972 0.166 2.929 8.240

Coefficients:

Estimate Std. Error t value Pr(>|t|)

(Intercept) 25.56296 22.81380 1.121 0.2675

modulation_indexT1theta 6.65707 3.31083 2.011 0.0494 *

modulation_indexT2theta -2.57820 2.61298 -0.987 0.3282

absolute_powerT1theta 0.84006 0.57220 1.468 0.1479

Age_at_T3 0.04446 0.03003 1.481 0.1445

---

Signif. codes: 0 ‘***’ 0.001 ‘**’ 0.01 ‘*’ 0.05 ‘.’ 0.1 ‘ ’ 1

Residual standard error: 4.434 on 54 degrees of freedom

Multiple R-squared: 0.1527, Adjusted R-squared: 0.08991

F-statistic: 2.432 on 4 and 54 DF, p-value: 0.05845

AIC = 180.53

Model 5 provided the best fit, and is described in the main manuscript.

1. Predicting Selective attention with 6- and 12-month variables as separate predictors, and controlling for age at outcome: theta modulation at 6 (modulation_indexT1theta) and 12 (modulation_indexT2theta) months, absolute theta power at 6 (absolute_powerT1theta) and 12 (absolute_powerT2theta) months, relative theta power at 6 (relative_powerT1theta) and 12 (relative_powerT2theta) months.

Model 1 Summary:

Call:

lm(formula = Visual_search ~ modulation_indexT1theta + modulation_indexT2theta +

absolute_powerT1theta + absolute_powerT2theta + relative_powerT1theta +

relative_powerT2theta + Age_at_T3, data = DB_clean_VSearch2)

Residuals:

Min 1Q Median 3Q Max

-2.31431 -0.80015 0.00237 0.51515 2.62851

Coefficients:

Estimate Std. Error t value Pr(>|t|)

(Intercept) -0.127823 7.027371 -0.018 0.986

modulation_indexT1theta 1.606055 1.059610 1.516 0.136

modulation_indexT2theta 0.039341 0.813657 0.048 0.962

absolute_powerT1theta -0.077906 0.190412 -0.409 0.684

absolute_powerT2theta 0.198405 0.205916 0.964 0.340

relative_powerT1theta -6.433910 4.582836 -1.404 0.167

relative_powerT2theta 3.328564 4.974850 0.669 0.507

Age_at_T3 0.005203 0.008811 0.591 0.558

Residual standard error: 1.276 on 46 degrees of freedom

Multiple R-squared: 0.1345, Adjusted R-squared: 0.002771

F-statistic: 1.021 on 7 and 46 DF, p-value: 0.4294

AIC = 33.66

Model 2 Summary:

Call:

lm(formula = Visual_search ~ modulation_indexT1theta + absolute_powerT1theta +

absolute_powerT2theta + relative_powerT1theta + relative_powerT2theta +

Age_at_T3, data = DB_clean_VSearch2)

Residuals:

Min 1Q Median 3Q Max

-2.30388 -0.81084 0.00174 0.51683 2.63066

Coefficients:

Estimate Std. Error t value Pr(>|t|)

(Intercept) -0.117576 6.949224 -0.017 0.987

modulation_indexT1theta 1.610538 1.044283 1.542 0.130

absolute_powerT1theta -0.078814 0.187462 -0.420 0.676

absolute_powerT2theta 0.197621 0.203085 0.973 0.335

relative_powerT1theta -6.430657 4.533446 -1.418 0.163

relative_powerT2theta 3.282944 4.832436 0.679 0.500

Age_at_T3 0.005242 0.008680 0.604 0.549

Residual standard error: 1.262 on 47 degrees of freedom

Multiple R-squared: 0.1344, Adjusted R-squared: 0.02394

F-statistic: 1.217 on 6 and 47 DF, p-value: 0.3147

AIC = 31.66

Model 3 Summary:

Call:

lm(formula = Visual_search ~ modulation_indexT1theta + absolute_powerT2theta +

relative_powerT1theta + relative_powerT2theta + Age_at_T3,

data = DB_clean_VSearch2)

Residuals:

Min 1Q Median 3Q Max

-2.36060 -0.73592 0.01553 0.52119 2.61073

Coefficients:

Estimate Std. Error t value Pr(>|t|)

(Intercept) -0.655505 6.771593 -0.097 0.923

modulation_indexT1theta 1.619625 1.035067 1.565 0.124

absolute_powerT2theta 0.163231 0.184284 0.886 0.380

relative_powerT1theta -6.718399 4.442890 -1.512 0.137

relative_powerT2theta 3.818826 4.621164 0.826 0.413

Age_at_T3 0.005312 0.008604 0.617 0.540

Residual standard error: 1.251 on 48 degrees of freedom

Multiple R-squared: 0.1312, Adjusted R-squared: 0.04068

F-statistic: 1.449 on 5 and 48 DF, p-value: 0.224

AIC = 29.86

Model 4 Summary:

Call:

lm(formula = Visual_search ~ modulation_indexT1theta + absolute_powerT2theta +

relative_powerT1theta + relative_powerT2theta, data = DB_clean_VSearch2)

Residuals:

Min 1Q Median 3Q Max

-2.44679 -0.80416 0.07416 0.52558 2.67648

Coefficients:

Estimate Std. Error t value Pr(>|t|)

(Intercept) 3.2026 2.5935 1.235 0.223

modulation_indexT1theta 1.6260 1.0285 1.581 0.120

absolute_powerT2theta 0.1740 0.1823 0.955 0.344

relative_powerT1theta -6.8982 4.4053 -1.566 0.124

relative_powerT2theta 3.9993 4.5827 0.873 0.387

Residual standard error: 1.244 on 49 degrees of freedom

Multiple R-squared: 0.1243, Adjusted R-squared: 0.05279

F-statistic: 1.738 on 4 and 49 DF, p-value: 0.1566

AIC = 28.29

Model 5 Summary:

Call:

lm(formula = Visual_search ~ modulation_indexT1theta + absolute_powerT2theta +

relative_powerT1theta, data = DB_clean_VSearch2)

Residuals:

Min 1Q Median 3Q Max

-2.38759 -0.80636 -0.02035 0.60220 2.75114

Coefficients:

Estimate Std. Error t value Pr(>|t|)

(Intercept) 4.0534 2.3976 1.691 0.0971 .

modulation_indexT1theta 1.6713 1.0247 1.631 0.1092

absolute_powerT2theta 0.2096 0.1772 1.183 0.2425

relative_powerT1theta -5.6255 4.1469 -1.357 0.1810

---

Signif. codes: 0 ‘***’ 0.001 ‘**’ 0.01 ‘*’ 0.05 ‘.’ 0.1 ‘ ’ 1

Residual standard error: 1.241 on 50 degrees of freedom

Multiple R-squared: 0.1107, Adjusted R-squared: 0.05731

F-statistic: 2.074 on 3 and 50 DF, p-value: 0.1155

AIC = 27.12

Model 6 Summary:

Call:

lm(formula = Visual_search ~ modulation_indexT1theta + relative_powerT1theta,

data = DB_clean_VSearch2)

Residuals:

Min 1Q Median 3Q Max

-2.62271 -0.76903 -0.09624 0.61754 2.65204

Coefficients:

Estimate Std. Error t value Pr(>|t|)

(Intercept) 5.458 2.091 2.610 0.0118 *

modulation_indexT1theta 1.905 1.009 1.888 0.0648 .

relative_powerT1theta -4.581 4.068 -1.126 0.2654

---

Signif. codes: 0 ‘***’ 0.001 ‘**’ 0.01 ‘*’ 0.05 ‘.’ 0.1 ‘ ’ 1

Residual standard error: 1.245 on 51 degrees of freedom

Multiple R-squared: 0.08578, Adjusted R-squared: 0.04993

F-statistic: 2.393 on 2 and 51 DF, p-value: 0.1016

AIC = 26.61

Model 7 provided the best fit and is described in the main manuscript.

1. Predicting Language with 6- and 12-month variables as separate predictors, and controlling for age at outcome: theta modulation at 6 (modulation_indexT1theta) and 12 (modulation_indexT2theta) months, absolute theta power at 6 (absolute_powerT1theta) and 12 (absolute_powerT2theta) months, relative theta power at 6 (relative_powerT1theta) and 12 (relative_powerT2theta) months.

Model 1 Summary:

Call:

lm(formula = Language ~ modulation_indexT1theta + modulation_indexT2theta +

absolute_powerT1theta + absolute_powerT2theta + relative_powerT1theta +

relative_powerT2theta + Age_at_T3, data = DB_clean_Language2)

Residuals:

Min 1Q Median 3Q Max

-1.9756 -0.5550 0.1194 0.5659 1.7983

Coefficients:

Estimate Std. Error t value Pr(>|t|)

(Intercept) -8.022791 5.190098 -1.546 0.1289

modulation_indexT1theta -0.618346 0.809151 -0.764 0.4486

modulation_indexT2theta -0.378951 0.570123 -0.665 0.5095

absolute_powerT1theta 0.091191 0.134310 0.679 0.5005

absolute_powerT2theta -0.178431 0.150436 -1.186 0.2415

relative_powerT1theta 3.482995 3.397592 1.025 0.3105

relative_powerT2theta -4.304248 3.221062 -1.336 0.1879

Age_at_T3 0.012622 0.006431 1.963 0.0556 .

---

Signif. codes: 0 ‘***’ 0.001 ‘**’ 0.01 ‘*’ 0.05 ‘.’ 0.1 ‘ ’ 1

Residual standard error: 0.9303 on 47 degrees of freedom

Multiple R-squared: 0.1714, Adjusted R-squared: 0.04803

F-statistic: 1.389 on 7 and 47 DF, p-value: 0.2322

AIC = -0.59

Model 2 Summary:

Call:

lm(formula = Language ~ modulation_indexT1theta + absolute_powerT1theta +

absolute_powerT2theta + relative_powerT1theta + relative_powerT2theta +

Age_at_T3, data = DB_clean_Language2)

Residuals:

Min 1Q Median 3Q Max

-2.04627 -0.56689 0.08764 0.57837 1.79904

Coefficients:

Estimate Std. Error t value Pr(>|t|)

(Intercept) -7.908998 5.157024 -1.534 0.1317

modulation_indexT1theta -0.720757 0.789714 -0.913 0.3660

absolute_powerT1theta 0.092792 0.133506 0.695 0.4904

absolute_powerT2theta -0.173061 0.149343 -1.159 0.2523

relative_powerT1theta 3.492833 3.377746 1.034 0.3063

relative_powerT2theta -4.248744 3.201202 -1.327 0.1907

Age_at_T3 0.012300 0.006376 1.929 0.0596 .

---

Signif. codes: 0 ‘***’ 0.001 ‘**’ 0.01 ‘*’ 0.05 ‘.’ 0.1 ‘ ’ 1

Residual standard error: 0.9249 on 48 degrees of freedom

Multiple R-squared: 0.1636, Adjusted R-squared: 0.0591

F-statistic: 1.565 on 6 and 48 DF, p-value: 0.1778

AIC = -2.08

Model 3 Summary:

Call:

lm(formula = Language ~ modulation_indexT1theta + absolute_powerT2theta +

relative_powerT1theta + relative_powerT2theta + Age_at_T3,

data = DB_clean_Language2)

Residuals:

Min 1Q Median 3Q Max

-2.05371 -0.52377 0.07227 0.58493 1.73243

Coefficients:

Estimate Std. Error t value Pr(>|t|)

(Intercept) -7.32622 5.06149 -1.447 0.1541

modulation_indexT1theta -0.75590 0.78392 -0.964 0.3397

absolute_powerT2theta -0.13664 0.13911 -0.982 0.3308

relative_powerT1theta 3.79334 3.33224 1.138 0.2605

relative_powerT2theta -4.62861 3.13752 -1.475 0.1465

Age_at_T3 0.01220 0.00634 1.925 0.0601 .

---

Signif. codes: 0 ‘***’ 0.001 ‘**’ 0.01 ‘*’ 0.05 ‘.’ 0.1 ‘ ’ 1

Residual standard error: 0.92 on 49 degrees of freedom

Multiple R-squared: 0.1552, Adjusted R-squared: 0.06902

F-statistic: 1.801 on 5 and 49 DF, p-value: 0.1302

AIC = -3.53

Model 4 Summary:

Call:

lm(formula = Language ~ absolute_powerT2theta + relative_powerT1theta +

relative_powerT2theta + Age_at_T3, data = DB_clean_Language2)

Residuals:

Min 1Q Median 3Q Max

-2.0074 -0.5367 0.0888 0.5731 1.8888

Coefficients:

Estimate Std. Error t value Pr(>|t|)

(Intercept) -7.266454 5.057557 -1.437 0.1570

absolute_powerT2theta -0.162344 0.136431 -1.190 0.2397

relative_powerT1theta 3.703711 3.328606 1.113 0.2712

relative_powerT2theta -4.400830 3.126419 -1.408 0.1654

Age_at_T3 0.012146 0.006336 1.917 0.0609 .

---

Signif. codes: 0 ‘***’ 0.001 ‘**’ 0.01 ‘*’ 0.05 ‘.’ 0.1 ‘ ’ 1

Residual standard error: 0.9194 on 50 degrees of freedom

Multiple R-squared: 0.1392, Adjusted R-squared: 0.07033

F-statistic: 2.021 on 4 and 50 DF, p-value: 0.1057

AIC = -4.49

Model 5 Summary:

Call:

lm(formula = Language ~ absolute_powerT2theta + relative_powerT2theta +

Age_at_T3, data = DB_clean_Language2)

Residuals:

Min 1Q Median 3Q Max

-1.88829 -0.57413 0.01713 0.54882 2.04668

Coefficients:

Estimate Std. Error t value Pr(>|t|)

(Intercept) -5.804182 4.895219 -1.186 0.241

absolute_powerT2theta -0.163589 0.136745 -1.196 0.237

relative_powerT2theta -3.001812 2.869124 -1.046 0.300

Age_at_T3 0.011874 0.006346 1.871 0.067 .

---

Signif. codes: 0 ‘***’ 0.001 ‘**’ 0.01 ‘*’ 0.05 ‘.’ 0.1 ‘ ’ 1

Residual standard error: 0.9215 on 51 degrees of freedom

Multiple R-squared: 0.1179, Adjusted R-squared: 0.06599

F-statistic: 2.272 on 3 and 51 DF, p-value: 0.09132

AIC = -5.15

Model 6 provided the best fit and is described in the main manuscript.

S10) Examining the linear assumptions of the theta modulation index

In order to evaluate whether the linear assumptions underlying the theta modulation index, we examined the fit of the linear model on data from a collapsed sample. Closely following the steps described by Braithwaite et al. (2020), we averaged data (absolute power in the theta frequency band) from each infant who provided a clean segment within each second of the video and modelled the relation with time for bins in which at least 5 infants contributed data (all but one bin for 6-month-olds, and all bins for 12 month-olds). We then examined the fit of the linear regression model.


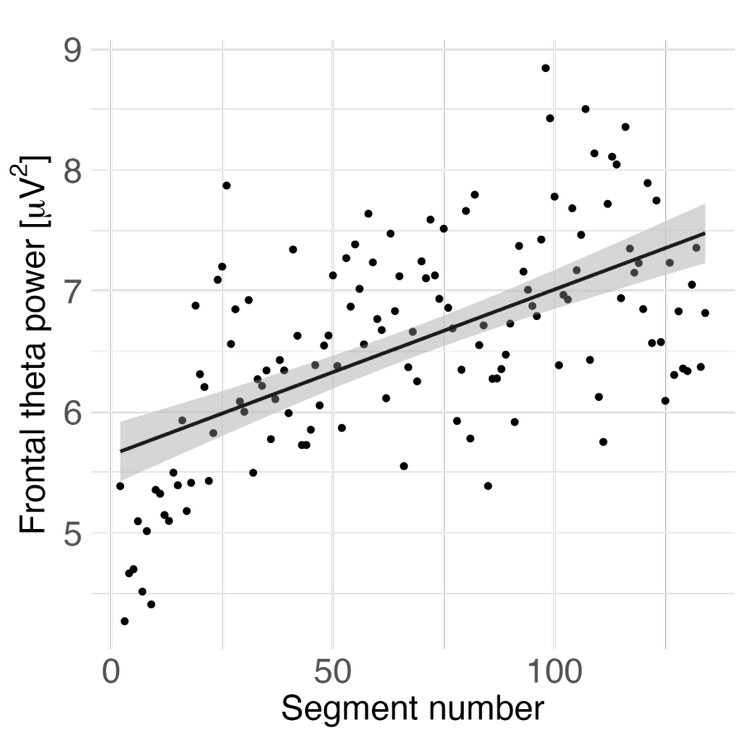
Figures S1 and S1 depict the data and fitted linear models for 6- and 12-month-olds.

Figure S1. Data from the collapsed 6-month-old sample, generated by averaging frontal theta power data from each infant who provided a clean segment within each second of the video and modelling the relation with time for bins with at least 5 segments of clean data, fitted with a linear model.

In both 6-month-olds (F(1, 131) = 70.53, p <0.001; adjusted R^2^ = 0.345, standardized β = 0.59, t(131) = 8.40, p <0.001) and 12-month-olds (F(1, 132) = 27.88, p < 0.001, adjusted R^2^ = 0.168, standardized β = 0.42, t(132) = 5.28, p < 0.001) the linear model fit well, supporting the assumptions underlying the theta modulation index.


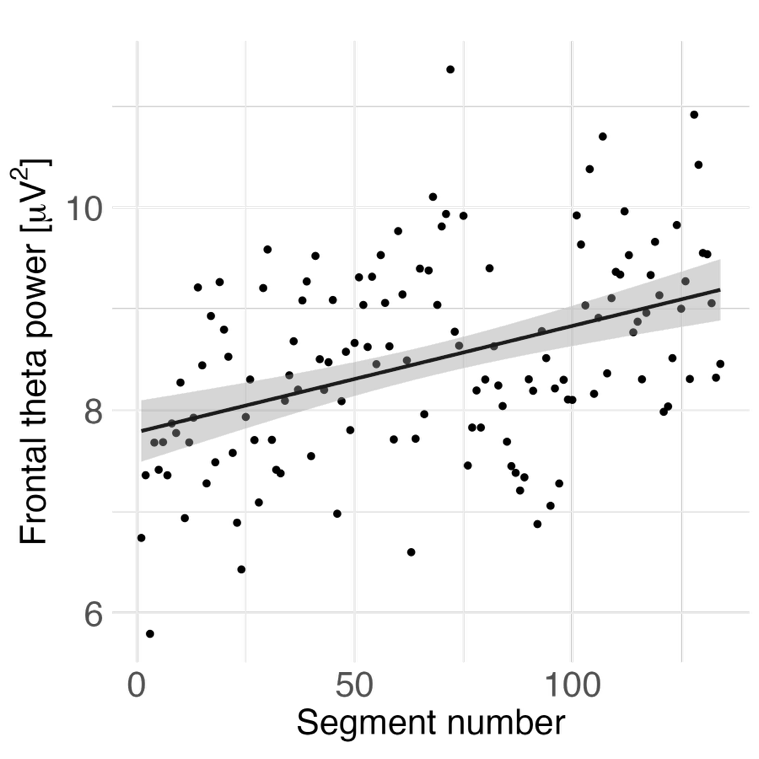


Figure S2. Data from the collapsed 12-month-old sample, generated by averaging frontal theta power data from each infant who provided a clean segment within each second of the video and modelling the relation with time for bins with at least 5 segments of clean data, fitted with a linear model.
